# Prefrontal projections to the bed nuclei of the stria terminalis modulate the specificity of aversive memories

**DOI:** 10.21203/rs.3.rs-4241372/v1

**Published:** 2024-10-21

**Authors:** Ryan T. Lingg, Shane B. Johnson, Dalton C. Hinz, Timothy D. Skog, Manuela Lizarazu, Sara A. Romig-Martin, Ryan T. LaLumiere, Nandakumar S. Narayanan, Jason J. Radley

**Affiliations:** 1 Department of Psychological and Brain Sciences, University of Iowa, Iowa City, IA, USA; 2 Interdisciplinary Neuroscience Program, University of Iowa, Iowa City, IA, USA; 3 Department of Neurology, Carver College of Medicine, University of Iowa, Iowa City, IA, USA; 4 Iowa Neuroscience Institute, University of Iowa, Iowa City, IA, USA

## Abstract

Generalizing aversive memories helps organisms avoid danger, whereas discriminating between dissimilar situations promotes opportunistic behaviors. We identified a novel pathway that controls the contextual specificity of memory consolidation of inhibitory avoidance learning. Optogenetic inhibition of the rostral medial prefrontal cortex (mPFC)-to-anteroventral bed nuclei of the stria terminalis (avBST) pathway after a single footshock exacerbated stress hormonal output, and 2 d later promoted generalization to a novel context. Rostral mPFC–avBST influences were directly mnemonic rather than associated with stress hormone increases, as adrenalectomy did not prevent such influences on generalization. We next observed that fear discrimination between novel and aversive contexts engaged activity along the rostral mPFC and avBST pathway. Finally, post-footshock optogenetic pathway excitation enhanced 2-d discrimination. These findings highlight a prefrontal pathway in which activity immediately after aversive experiences promotes mnemonic discrimination between threatening and non-threatening contexts and may be importance for understanding trauma generalization in psychiatric illnesses.

## INTRODUCTION

Forming memories of aversive experiences helps animals anticipate and respond accordingly to future threats using a range of defensive behaviors. The strength of the memory determines the degree to which the animal expresses such behaviors when faced with a similar situation. By contrast, memory specificity—the degree to which a memory is recalled in circumstances of varying similarity—optimizes how prior knowledge is used to minimize potential risks while maximizing potential reward. Indeed, whereas fear memory generalization provides adaptive benefit for ensuring defensive behaviors in similar situations that may turn out to be threatening, memory discrimination provides adaptive benefits by ensuring that animals do not display such behaviors in distinct situations that could result in potential missed opportunities.

During memory consolidation after an aversive event, memory specificity may be influenced by the severity of the threat encountered, prior experience, and accompanying changes in stress hormone output.^1–3^ How well the memory of an experience is consolidated and the specificity of the associated details are determined by amygdaloid-hippocampal network activity. Whereas basolateral amygdala activation is important for encoding certain types of threats and consolidating emotional experiences,^4–12^ hippocampal processing is critical for encoding and retrieval of contextual memories and discrimination.^13–19^ However, less is known regarding the mechanisms that shape memory specificity in the aftermath of an aversive experience.

The prelimbic region (PL) of the medial prefrontal cortex (mPFC) is important in regulating aspects of aversive memory, including during acquisition and subsequent recall.^20–27^ The mPFC projects broadly to a number of regions that may modulate different aspects of learned fear through output to the amygdaloid complex,^24, 27–31^ periaqueductal gray (PAG),^22, 32, 33^ and bed nuclei of the stria terminalis (BST). ^34–36^ Yet, these memory processing features of the mPFC are difficult to disentangle from its more commonly known role in situationally adjusting behavioral strategies according to environmental demands,^37–40^ such as when responding to a threat or coping with stress.^41, 42^ The rostral (r) PL projection to the anteroventral (av) BST coordinates acute behavioral and neuroendocrine responses to a diverse array of challenges,^43–46^ and the avBST demonstrates the capacity to modulate how strongly memories are consolidated after an aversive event through its projections to hypothalamo-pituitary-adrenal (HPA) effector neurons in the paraventricular nucleus of the hypothalamus and ventrolateral PAG.^47–50^ Collectively, these findings implicate rPL projections to the avBST as a novel candidate for modulating memory strength and specificity in the aftermath of an aversive experience.

Here, we evaluated involvement of the rPL–avBST pathway in aversive memory consolidation using the inhibitory avoidance (IA) discrimination learning task in rats developed by Roozendaal and colleagues (see Materials and Methods),^51^ and examined whether the involvement of this pathway was directly mnemonic rather than associated with its influence over HPA glucocorticoid hormones. Surprisingly, our data revealed activity changes along the rPL–avBST pathway after IA discrimination learning that distinguish safe from aversive contexts, as well as a bi-directional role of the rPL–avBST pathway in modulating memory specificity while leaving memory strength unchanged. These results provide a new conceptualization of memory consolidation that involves rPL–avBST activity changes after aversive experiences that are important for dissociably influencing the contextual specificity or an aversive event from well it is remembered.

## RESULTS

### Functional anatomical characterization of prefrontal–BST projections in IA discrimination

**Figure 1.**
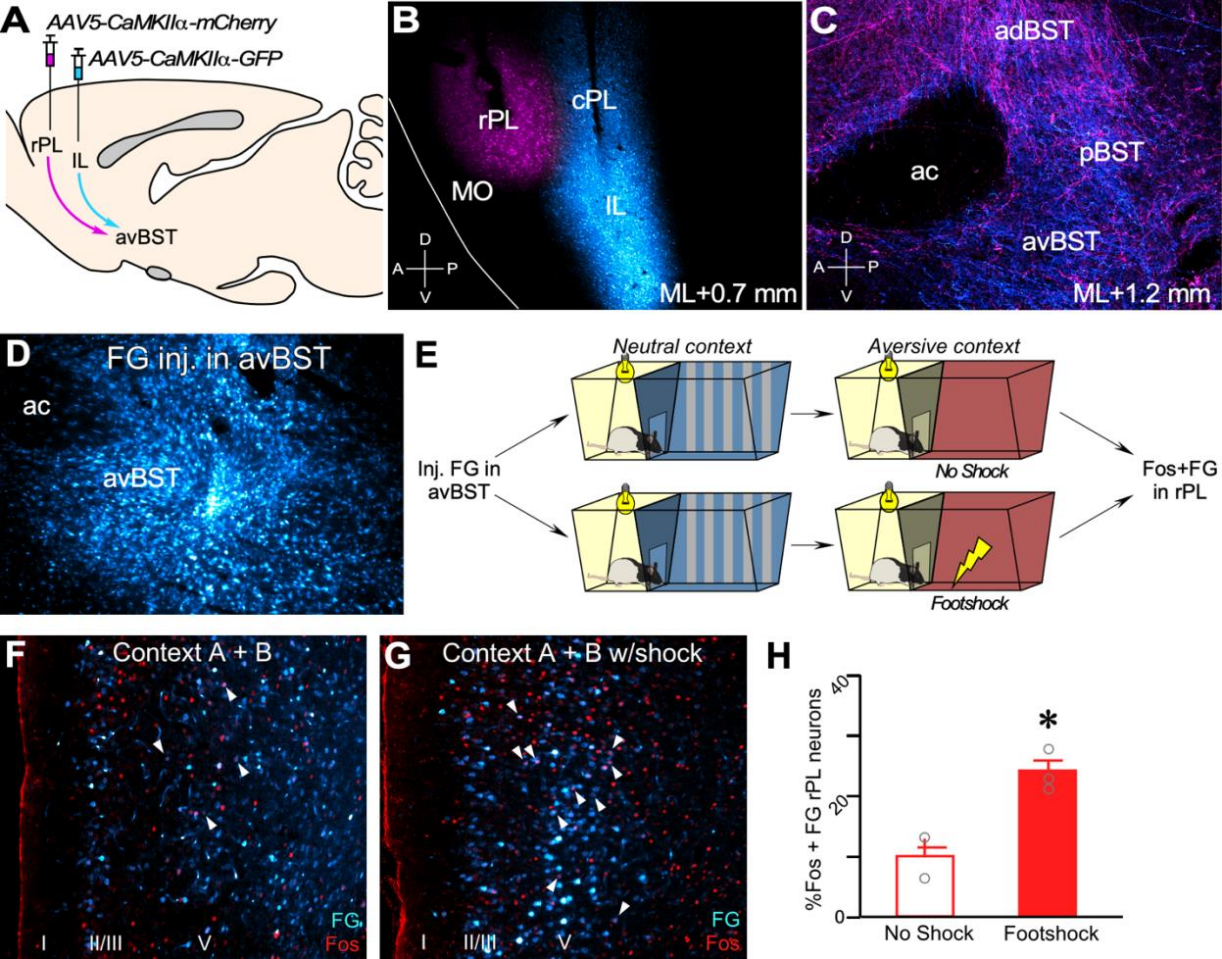
Functional neuroanatomical characterization of prefrontal projections to bed nuclei of the stria terminalis (BST). (**A**) Midsagittal diagram depicting AAV5-CaMKIIα microinjections made in rostral prelimbic cortex (rPL; mCherry, magenta) and infralimbic cortex (IL; GFP, cyan) prefrontal subfields in the same rat. (**B**) Epifluorescent parasagittal (mediolateral, ML, 0.7 mm) image showing the distribution of mCherry and GFP labeling of injection sites in rPL and IL. cPL, caudal PL; MO, medial orbitofrontal cortex. (**C**) Parasagittal (ML 1.2 mm) confocal image illustrates the distribution of mCherry- and GFP-labeled terminal fields in BST. ac, anterior commissure; adBST, anterodorsal BST; avBST, anteroventral BST; pBST, posterior BST. (**D**) Representative case of a microinjection of Fluoro-Gold (FG) centered in the avBST. (**E**) Diagram of behavioral procedures for the Fos experiment, involving sequential exposures to neutral and aversive contexts to groups of rats, with one group receiving a footshock (0.8 mA, 1 s) in the aversive context. (**F, G**) Rats were perfused 90 min later and evaluated for dual immunolabeling for FG (cyan) and Fos (red). Example images of rPL in groups of rats receiving no shock (**F**) or footshock (**G**). Arrowheads point to examples of dual-labeled neurons. (**H**) Histogram showing that rats in the footshock group displayed increased activation in the percentage of the total number of Fos-labeled avBST-projecting rPL neurons. Mean ± SEM; n = 3 per group; *, *p* < 0.05.

Both the mPFC and avBST are reliably implicated in stress- and avoidance-related behaviors.^52–54^ We neuroanatomically characterized PL projections, focusing specifically on innervation of the BST, and compared them with projections to the BST from the adjacent infralimbic cortex (IL).^55–58^ We microinjected adeno-associated virus (AAV5) expressing either mCherry or green fluorescent protein (GFP), each under the CAMKIIα promoter, into PL and IL of adult male rats (Fig. 1, A–B; Fig. S1). Viral expression was evaluated based on PL and IL cytoarchitectonic parcellations and adjacent subfields.^59–63^ Because prior work showed that rostral and ventral aspects of PL exhibit moderate-to-dense BST innervation versus that in more dorsal and caudal regions,^58, 62, 64–66^ we targeted AAV injections to rostrally-situated layer V/VI glutamatergic projection neurons in PL. AAV injections in rPL revealed innervation in the lateral subdivisions of BST (Fig. S2), including subcommissural, dorsomedial, fusiform, and rhomboid subdivisions described by Swanson and colleagues.^67–70^ By contrast, AAV injections in IL produced more widespread innervation throughout BST, including both medial and lateral regions of the anterior and posterior quadrants (Fig. 1C; Fig. S2).^56, 64^

To further investigate the anterograde pathway data supporting a projection from rPL to the avBST, we microinjected the retrogradely transported AAV2-retro-GFP ^71^ into the avBST and analyzed GFP expression patterns throughout mPFC (Fig. S3). In line with our anterograde tracing experiments, we observed a paucity of retrograde expression of GFP at caudal levels of PL, with increasing expression density at rostral levels of PL, particularly in deeper layers (Fig. S3). These results were further supported by examination of retrograde accumulation of the fluorescent dye, Fluoro-Gold (FG), following injections made in the avBST (Fig. 1D; Fig. S3).

We next evaluated whether these retrogradely-labeled PL neurons are engaged after IA training. Rats with FG injections in the avBST were placed in the brightly-lit side of a standard IA apparatus, which is a trough-shaped box segmented with one side containing an illuminated white plastic bottom and the other a darkened stainless-steel bottom, and a retractable stainless-steel door separating each compartment. Rats were allowed 1 min to freely explore this IA apparatus, designated as the *neutral context*, and they were next transferred to the brightly lit side of a second, contextually modified IA chamber containing strips of white tape spaced 2 in apart. Upon entering the darkened compartment, the retractable door was closed, and 20 s later rats received a single footshock (Fig. 1E), hence designating this as the aversive *context*.^51, 72–74^ A second control group of rats was exposed to the same IA contexts as described, but they did not receive a footshock. Ninety minutes following IA training, both groups of rats were perfused. rPL sections were prepared using dual immunohistochemistry for FG and the immediate early gene transcription factor, c-Fos, to evaluate functional activation in the rPL–avBST pathway (Fig. 1, F–G). As both groups were exposed to both IA contexts, rats that received the footshock versus the non-shocked group had increased c-Foslabeled cells and c-Fos colocalization with FG-labeled neurons (Fig. 1, H–I), suggesting that post-training activation in the rPL–avBST pathway may be an important event in the consolidation of an aversive experience.

### Inhibiting the rPL–avBST pathway augments HPA activation and shifts memory consolidation toward generalization.

**Figure 2.**
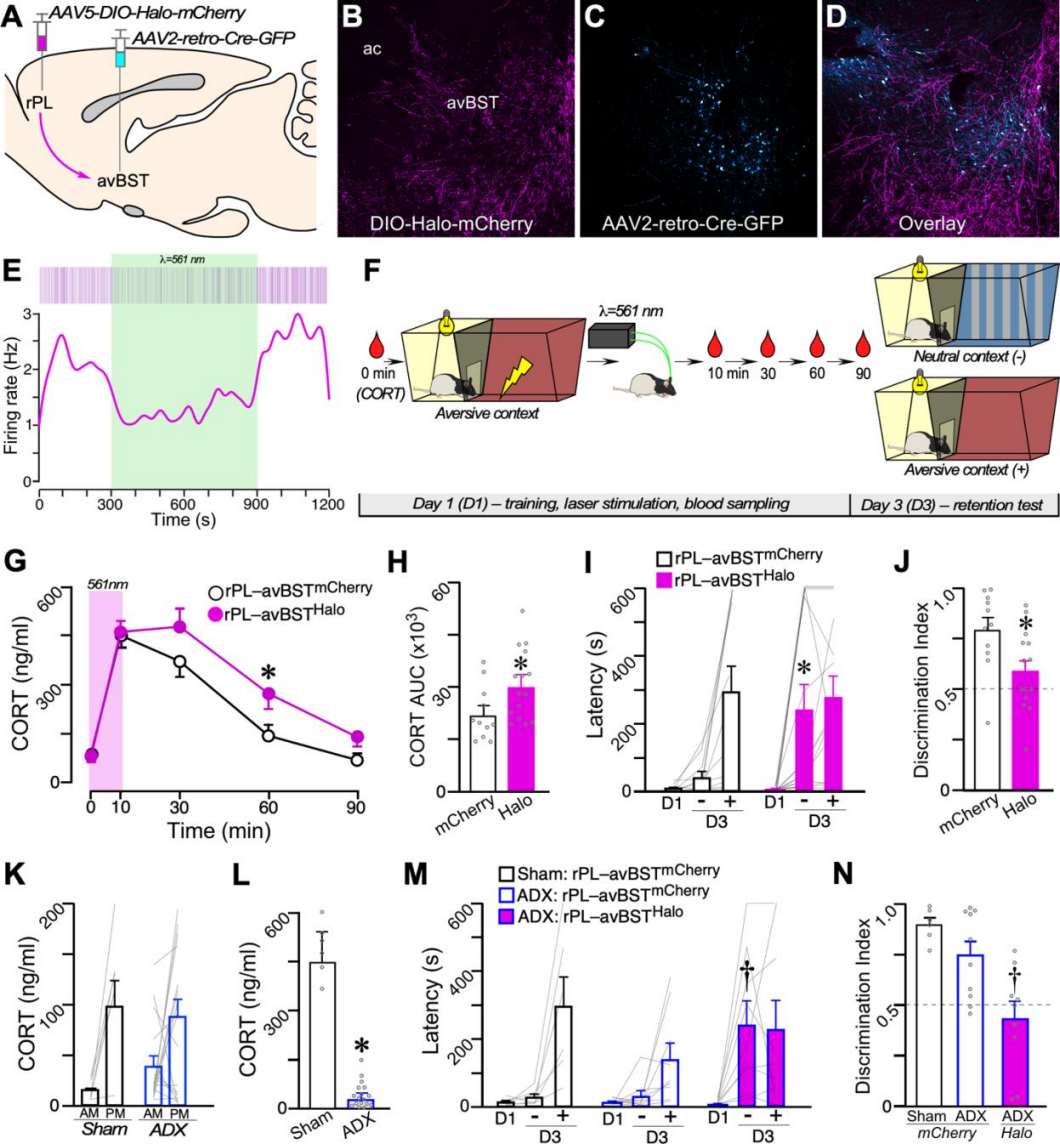
Post-training inhibition of rPL–avBST activity promotes fear generalization in a glucocorticoid-independent manner. (**A**) Diagram of the viral strategy used for targeting the rPL–BST pathway, which involved microinjections of AAV2-retro-Cre-GFP in the avBST and either cre-dependent AAV5-DIO-Halo-mCherry or control in the rPL. Optical fibers were placed dorsal to avBST to selectively inhibit the rPL–avBST pathway. (**B–D**) Representative fluorescent images illustrate dense viral expression of rPL axonal input to the avBST. ac, anterior commissure. (**E**) Electrophysiological verification of opsin functionality in the avBST; 561-nm laser stimulation (green shaded region) was applied for 10 min. (**E, *top***) Randomized raster of unit activity (top) prior to, during, and post-laser stimulation. Z-scored unit activity (bottom) across the entire recording session. (**F**) Illustration of IA discrimination task and timeline of the behavioral procedures. Red droplets indicate time points of blood collection for the corticosterone (CORT) radioimmunoassay. (**G**) CORT responses on testing day 1, prior to (0 min) and after footshock (10, 30, 60, 90 min) revealed overall significant increases across time and a selective increase at 60 min for rPL–avBST^Halo^-expressing animals. (**H**) Integrated CORT responses (Area under curve, AUC) are significantly increased (*p* < 0.05) in rPL–avBST^Halo^, compared to rPL–avBST^mCherry^ groups. (**I**) Avoidance latencies across day 1 (D1) and day 3 (D3) in neutral (−) and aversive (+) contexts revealed a significant increase in the latency to avoid the neutral context in rPL–avBST^Halo^ relative to rPL–avBST^mCherry^ rats. (**J**) Discrimination index values (i.e., latency aversive/ [latency neutral + aversive contexts]) revealed a significant decrease in rPL–avBST^Halo^, as compared to rPL–avBST^mCherry^ rats. n = 15, rPL–avBST^Halo^; n = 11, rPL–avBST^mCherry^. *, *p* < 0.05. (**K**) Some rats received adrenalectomy (ADX) with CORT (25 mg/dl) replacement in their drinking water for 14 d to prevent the footshock-induced increase in CORT, while minimally disrupting the basal CORT rhythm, as based on blood sampling near the circadian trough (AM, 0500) and peak (PM, 1700). (**L**) Following completion of behavioral training and testing, CORT was removed from drinking water of the ADX group for 7 d, followed by 30 min exposure to restraint stress for verification of ADX efficacy. (**M**) Avoidance latencies during training on D1 remained the same across all treatments; however, the ADX: rPL–avBST^Halo^ group displayed increased retention latencies to the neutral context (−) on D3 as compared to sham and ADX groups receiving only laser stimulation. (**N**) Discrimination indices were significantly decreased in the ADX: rPL–avBST^Halo^ but not in ADX: rPL–avBST^mCherry^ group, each versus sham controls. n = 6, Sham: rPL–avBST^mCherry^; n = 9, ADX: rPL–avBST^mCherry^; n = 9, ADX: rPL–avBST^Halo^. *, *p* < 0.05 relative to Sham. †, *p* < 0.05, relative to Sham: rPL–avBST^mCherry^ and ADX:rPL–avBST^mCherry^. Also see Supplementary Materials, Statistical Analyses.

BST inactivation immediately after IA training enhances consolidation through disinhibiting the HPA axis and potentiating corticosterone output.^47^ However, exogenously increasing glucocorticoid levels after acquisition has been shown to increase memory strength and generalization.^1, 2, 10, 75–78^ Based upon these prior findings, we compared whether rPL input to the avBST modulates memory strength versus memory specificity and how these processes depend on glucocorticoids. We used an intersectional optogenetic approach to inhibit avBST-projecting neurons in rPL immediately following IA training, within the timeframe known to be necessary for the glucocorticoid-dependent enhancement of memory consolidation. ^5, 10, 47, 79, 80^ We injected a retrogradely transported AAV encoding Cre recombinase in the avBST,^71^ and we injected a Cre-dependent AAV in the rPL to express inhibitory opsin-enhanced halorhodopsin 3.0 (Halo) fused to mCherry (rPL–avBST^Halo)^. Control rats received the same procedures, except that the AAV injections in the rPL region expressed mCherry instead of Halo (rPL–avBST^mCherry^)(Fig. 2, A–D).^81^ Both groups were implanted with optical fibers bilaterally over the avBST for projection-specific rPL–avBST inhibition.

We confirmed the efficacy of our approach by performing neuronal recordings in the avBST in vivo using an optrode consisting of a 16-wire microelectrode containing one optical fiber. Photoinhibition of rPL fibers in BST with 561-nm laser light reliably decreased neuronal activity over the entirety of the 10-min stimulation period (Fig. 2E). Previous work using the same laser during a 15-min stimulation period demonstrated no effect on neuronal responses in the avBST of control rats not expressing opsin,^44, 47^ indicating that illumination alone does not appreciably alter neural activity.

Two days prior to training in the IA discrimination learning task, all rats were fitted with indwelling jugular catheters for repeated blood sampling to assess levels of corticosterone (CORT, i.e., the rodent analog of cortisol). During IA training (day 1), a baseline blood sample was collected, and then rats were placed in the brightly lit compartment of the aversive context. Upon entering the darkened compartment, the retractable door was closed, and rats received a single footshock (Fig. 2F). Next, rats were placed back into their home cage and received constant 561-nm laser illumination for 10 min to inhibit the rPL–avBST pathway. The timing of circuit inactivation immediately following training is based on extensive evidence from us and others showing that this is a critical window for altering memory consolidation.^5, 10, 47, 79, 80^ Repeated blood sampling ensued following laser illumination (i.e., 10 min post-footshock), and at 30-, 60-, and 90-min intervals after the footshock.

On day 3, we assessed retention latencies for rPL–avBST^Halo^ and rPL–avBST^mCherry^ groups by placing rats back in the lit compartment of the aversive context and a similar, neutral context (as described above), in a counterbalanced manner. The retractable door remained open throughout, and rats’ latencies to enter the darkened compartment were used as an index of memory to the aversive context from training on day 1. Whereas high retention latencies to the aversive context is taken as a measure of memory strength, high latencies to the neutral context indicates fear generalization from the aversive context. We histologically verified that viral and optical fiber placements were appropriately placed in the avBST and that viral co-expression was distributed in rostral aspects of the mPFC (Fig. S4).

Radioimmunometric analysis of plasma CORT levels following a single footshock in the aversive context revealed post-training increases in both the rPL–avBST^mCherry^ and rPL–avBST^Halo^ groups (Fig. 2G). rPL–avBST^Halo^ rats displayed prolonged increases in CORT relative to rPL–avBST^mCherry^ controls, extending through 60 min post-training (Fig. 2G). Assessment of total CORT levels across the entire pre- and post-training period was measured as the area under the curve (AUC), confirming that rPL–BST^Halo^ rats displayed overall enhancements in CORT compared with the rPL–BST^mCherry^ group (Fig. 2H).

During retention testing on day 3, rPL–BST^mCherry^ rats successfully discriminated between the neutral and aversive contexts, displaying low and high latencies, respectively.(Fig. 2I). By contrast, rPL–BST^Halo^ rats displayed similarly high latencies in both contexts, suggesting that they generalized to the novel context and showed reduced memory specificity (Fig. 2I). Strikingly, rPL–avBST pathway inhibition had no effect on memory strength, as latencies in the aversive context in the rPL–avBST^Halo^ rats were comparable to those of rPL–BST^mcherry^ counterparts (Fig. 2I). Assessment of each group’s discrimination index (i.e., latency aversive context/ [latency aversive + latency neutral contexts]), further illustrated discrimination impairments in rPL–BST^Halo^ rats relative to their rPL–BST^mCherry^ counterparts (Fig. 2J). These results indicate that post-training inhibition of the rPL–avBST pathway augmented adrenocortical output and promoted a shift in memory specificity toward generalization, albeit without altering memory strength for the aversive context itself.

Since rPL–avBST inhibition enhanced post-training levels of CORT and subsequent generalization, we addressed whether the mnemonic effects of circuit inactivation were a secondary consequence of endogenous increases in glucocorticoids. This is based on evidence that exogenous CORT administration promotes fear generalization.^1, 2, 75–78^ We first verified that increasing post-training levels of CORT accounted for decreased memory specificity. We found that systemic post-training injections of CORT in the IA discrimination learning task (3 mg/kg, intraperitoneal; IP) enhanced generalization 2 d later by increasing retention latencies to the neutral context relative to vehicle-injected rats (Fig. S5).

If rPL–avBST inhibition promotes generalization by increased CORT levels after IA training, then we reasoned that inhibiting this pathway in rats lacking adrenal glands should prevent generalization. Rats expressing only Halo or mCherry in the rPL–avBST pathway received an adrenalectomy (ADX); control rats received a sham surgical procedure. Rats with ADX were administered CORT in drinking water (25 mg/dl) for a 14-d period prior to IA training; drinking behavior recapitulates natural diurnal variations in plasma CORT levels.^82^ Prior to IA training, we compared AM and PM levels of CORT in ADX and sham rats following serial blood collection from the tail vein, to verify the fidelity of the CORT replacement procedure (Fig. 2K). After IA training and testing were completed, we confirmed the efficacy of the ADX procedure by determining that there was a lack of CORT in ADX rats as compared with sham controls at the end of a 30-min period of restraint (Fig. 2L).

Groups of sham + rPL–avBST^mCherry^, ADX + rPL–avBST^mCherry^, and ADX + rPL–avBST^Halo^ rats were subjected to IA training, immediately followed by 10 min of 561-nm laser illumination in avBST. During IA training, both sham and ADX groups readily entered the darkened compartment of the aversive context (Fig. 2M). During testing on day 3, as expected the sham + rPL–avBST^mCherry^ group displayed high retention latencies in the aversive context and low retention in the neutral context (Fig. 2M). ADX + rPL–avBST^mCherry^ rats exhibited similarly high levels of discrimination, i.e., high and low retention latencies to the aversive and neutral contexts, respectively (*p* < 0.05 for each)(Fig. 2M). By contrast, ADX + rPL–avBST^Halo^ rats were unable to discriminate between the IA aversive and neutral contexts (respective mean latencies of 245 s and 232 s; *p* = 0.8)(Fig. 2M).

ADX + rPL–avBST^Halo^ rats displayed significantly higher retention latencies in the neutral context than mCherry controls regardless of adrenal status (8.8- and 7.9-fold increases relative to sham and ADX groups, respectively). Yet, retention in the aversive context between ADX + rPL–avBST^Halo^ and ADX + rPL–avBST^mCherry^ or sham + rPL–avBST^mCherry^ groups did not differ (*p* = 0.4 and 0.6, respectively)(Fig. 2M). Finally, discrimination indices were significantly lower in the ADX + rPL–avBST^Halo^ group relative to both mCherry control groups (by 52% in sham and 44% in the ADX groups)(Fig. 2N). Together, our results indicate that stress-related increases in glucocorticoids are capable of, but are not necessary for, promoting fear generalization after IA learning, and that they dissociate the effects of rPL–avBST pathway inhibition on memory generalization from accompanying increases in adrenocortical output.

### avBST activity correlates with memory specificity in the IA discrimination task.

**Figure 3.**
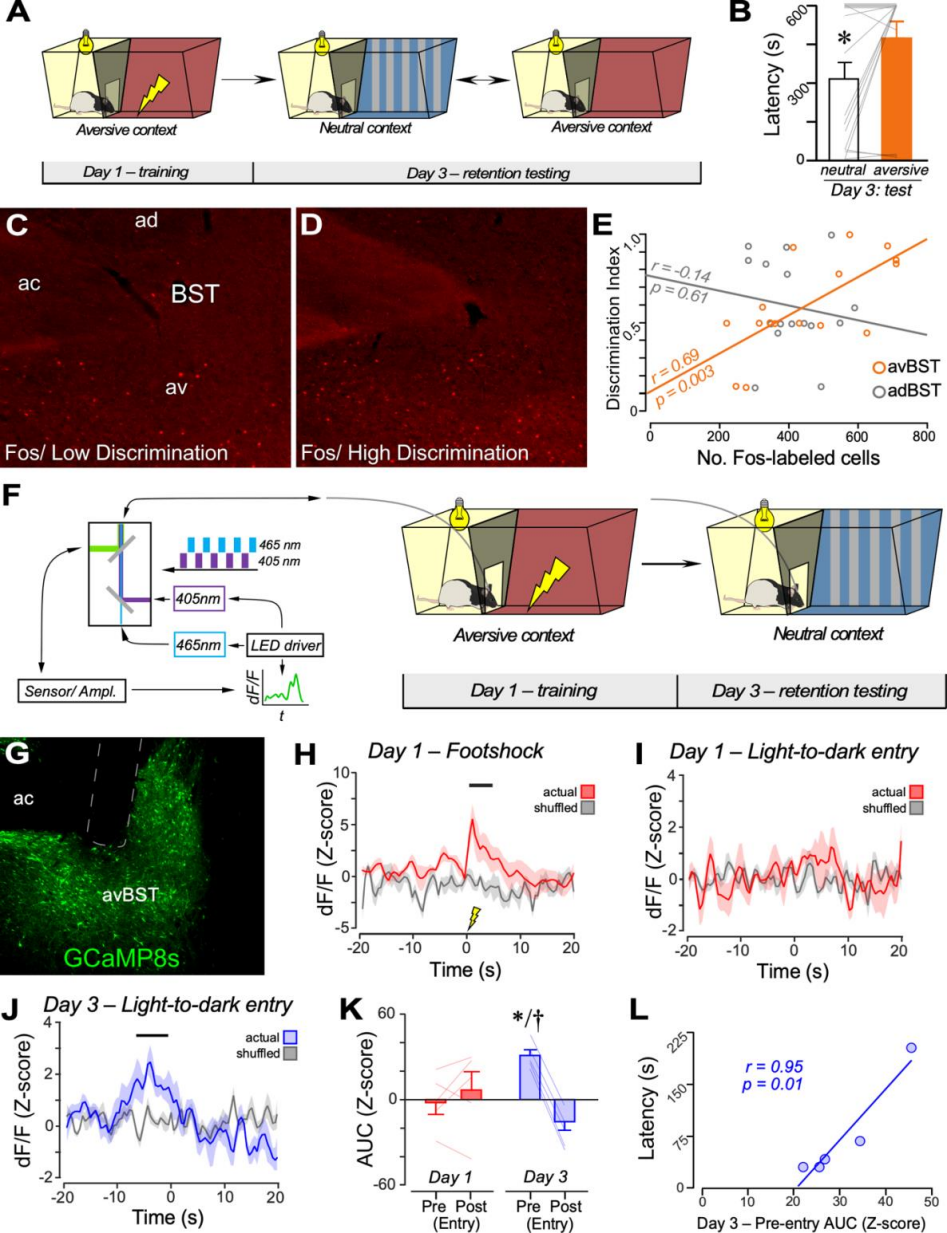
avBST calcium transients correlate with IA training and testing. (**A**) llustration of IA discrimination task and timeline of behavioral procedures. (**B**) On Day 3, rats show higher retention latencies to the aversive context relative to the neutral context. n = 16, *p* < 0.05. (**C, D**) Darkfield images showing examples of Fos immunoreactive cells in BST in cases that showed low (**C**) and high (**D**) discrimination indices. ac, anterior commissure; ad, anteroventral subdivision of BST; av, anteroventral subdivision of BST. (**E**) Scatter plot displays the number of Fos-labeled cells in the avBST (red) and adBST (gray) as a function of discrimination indices (i.e., latency training/[latency training + latency novel contexts]) for each rat. There was a strong positive correlation between Fos in the avBST, but not adBST, and discrimination indices. (**F**) Schematic of IA chamber in which avBST^GCaMP8s^ rats are placed for fiber photometry recordings during training and testing. Diagram (left) depicts 465-nm LED pulses for excitation of GCaMP8s interleaved with 405-nm isosbestic excitation to control for movement artifacts. Changes in fluorescence were collected, amplified, quantified (dF/F Z-score), and aligned to either footshock onset or latency to enter darkened compartment of IA chamber. (**G**) Exemplar from the avBST showing site of optical fiber placement and GCaMP8s expression. Dashed line, site of optical fiber placement. (**H–J**) Mean ± SEM for fluorescence traces in the avBST aligned to the onset of specific behavioral events (time 0), with shuffled data (gray) obtained by randomly resampling from dF/F values across each event. Significant increases (95% CI, black horizontal bar) in avBST activity were evident in response to footshock (**H**) in the aversive context on training day 1, and prior to entry from the light into the darkened compartment of the neutral context (**J**) on testing day 3. (**K**) AUC values on day 1 and day 3 during exposure to the neutral context is shown for each animal at 20-s intervals surrounding transitioning from the light (Pre) to the darkened compartment (Post) of IA chambers. On Day 3, pre-entry AUC values were significantly higher than post-entry into in the novel IA chamber (*, *p* < 0.05), and higher than pre-entry on day 1 (†, *p* < 0.05). (**L**) Scatter plot of pre-latency AUC values for day 3 and retention latency, each recorded during exposure to the neutral context. As latency increased, AUC values prior to latency also increased (*p* < 0.05). n = 5. Also see Supplementary Materials, Statistical Analyses.

We next considered whether changes in the avBST activity may confer evidence for the involvement of the avBST in discrimination learning. As a first step, we subjected rats to the IA discrimination task, involving exposure to the aversive context and footshock on training day 1. On day 3, we correlated how well rats discriminated between aversive and neutral contexts, as a function of neuronal activation in the avBST, using Fos immunohistochemistry (Fig. 3A). As expected, rats had significantly higher retention latencies in the aversive context compared to the neutral context (Fig. 3B). The number of Fos-immunoreactive cells in the avBST at 90 min post-testing showed a high positive correlation with the rats’ discrimination indices (Fig. 3, C–E). By contrast, we did not observe any correlation between the number of Fos-activated cells in the anterodorsal BST and discrimination indices in the same group of rats (Fig. 3E).

As a follow up, we evaluated avBST activity in the IA discrimination task utilizing fiber photometry to measure neuronal activity via calcium (Ca^2+^) levels. Rats received virus injections in the avBST to express the genetically encoded Ca^2+^ reporter, GCaMP8s, under the control of a synapsin promoter, and were implanted with optical fibers in the same region (Fig. 3, F–G). Photometry recordings of changes in avBST Ca^2+^ activity were used as an index of population activity during IA discrimination training and testing (Fig. 3, F–G). During IA training on day 1, whereas the footshock provoked a significant increase in Ca^2+^ activity in the avBST (Fig. 3H), we did not observe any Ca^2+^ activity changes during the 20-s time period between rats’ entry in the darkened compartment until footshock (Fig. 3I). On day 3, rats were tested in the neutral context, avBST activity was recorded for 10 min, and latencies to enter the darkened compartment were recorded. avBST activity increased prior to transitioning from the bright to the darkened compartment of the neutral context (Fig. 3J). This level of activity was not evident on the first day of IA training (Fig. 3K). Strikingly, we observed a significant correlation between retention latencies and avBST activity (20 s prior to entry; *r* = 0.95, *p* = 0.01) (Fig. 3L).

These data suggest that increased avBST activity may underlie entry into, versus avoidance of, the darkened compartment, as such increases occurred immediately prior to rats’ movements toward the darkened compartment of the neutral context after training in the aversive context. Yet, these data do not rule out the possibility that increased avBST activity may have been due instead to other factors related to the IA discrimination task, e.g., motivational properties associated with rats’ preferences for darker environments such as the darkened compartment in the neutral context. Thus, we performed an additional experiment in which rats were placed in a three-compartment apparatus with each end distinguished by bright or darkened lighting and contextually distinct features, and a middle compartment separating the two (Fig. S6). We recorded avBST Ca^2+^ transient activity while rats freely explored this chamber. Analyses of changes in avBST activity revealed nominal decreases in Ca^2+^ transients when rats transitioned from the middle compartment to the light or darkened compartments (Fig. S6). Nevertheless, this pattern of activity differed from the increase observed between the transition from the light to dark compartment in the neutral context during retention testing. Thus, the avBST activity increases observed between in the transition between light and darkened compartments in the neutral context on day 3 of testing more likely reflect discrimination rather preference for the darkened environment or exploration.^83^

### Prefrontal neuronal signatures of fear discrimination.

**Figure 4.**
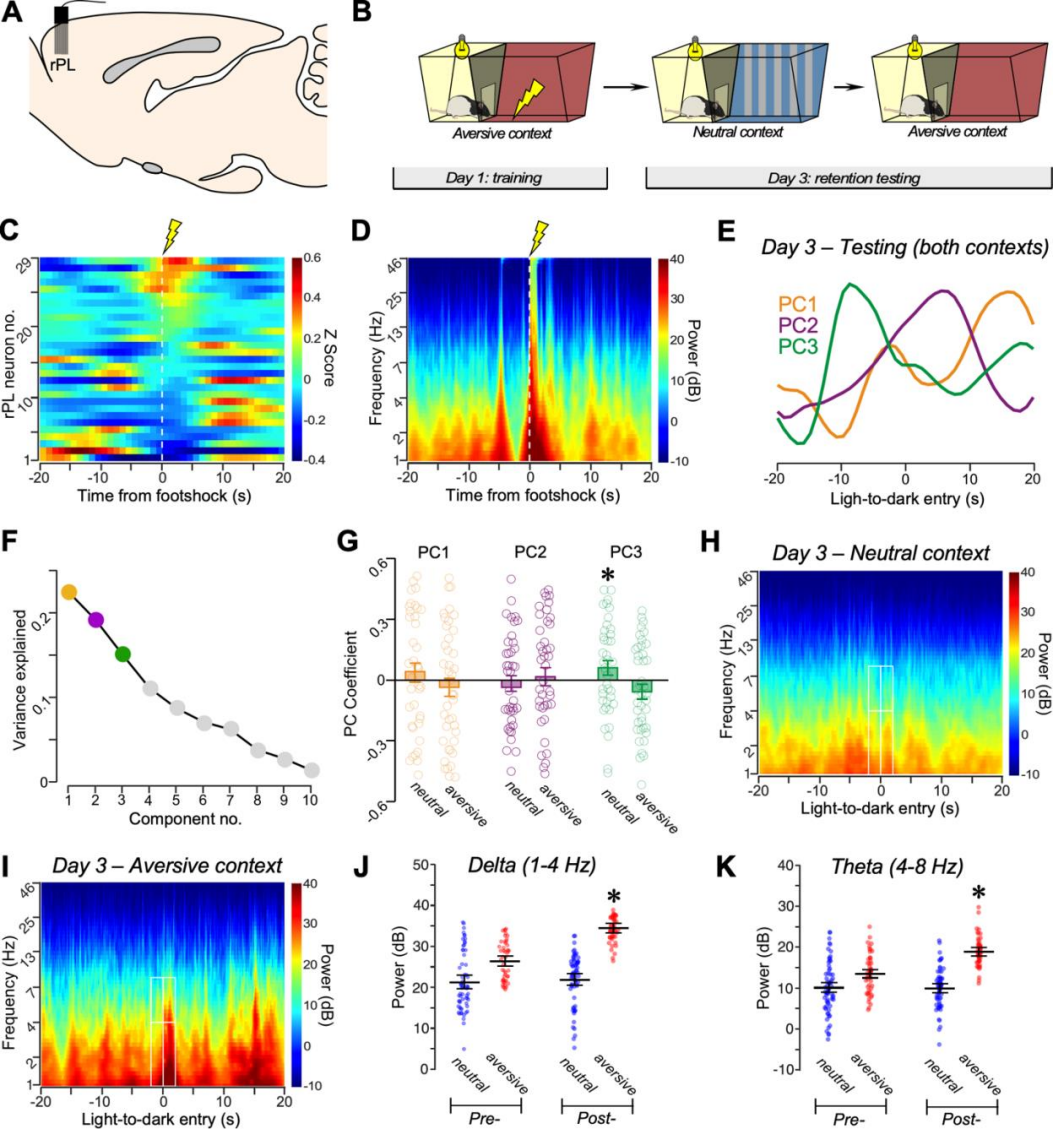
rPL neuron ensembles are engaged during IA training. (**A**) Schematic depicting 16-wire microelectrode array implantation in rPL. (**B**) Behavioral procedures for IA discrimination training on day 1 and retention testing on day 3 in aversive and neutral contexts, during simultaneous rPL recording. (**C**) Peri-event time histograms of 29 rPL units during IA training aligned to the onset of footshock (0 s; n = 29 units, n = 4 rats). (**D**) From 16 electrodes in the same four rats, time-frequency spectrograms revealed broadband modulation of activity in local field potentials (LFPs) in response to footshock during training on day 1. (**E**) Principal component (PC) analysis of neuronal ensembles on day 3 of retention testing. PCs are derived from rats’ latencies to enter to the dark compartment in neutral (42 neurons) and aversive (30 neurons) contexts. (**F**) Three PCs accounted for 22% (PC1), 19% (PC2), and 15% (PC3) of the variation in unit activity. (**G**) PC3, but not PC1 or PC2, coefficient values were increased in the neutral relative to aversive context. *, *p* < 0.05. (**H, I**) Spectrograms of LFPs in the neutral (**H**) and aversive (**I**) contexts on testing day 3. White boxes represent time-frequency regions-of-interest for analyses for delta (1–4 Hz) and theta (4–8 Hz) frequency ranges in (**J**) and (**K**), respectively, which revealed a main effect of context, a main effect of the 2-s post- versus 2-s pre-transition from light to dark compartments in each context, and a significant interaction. *, *p* < 0.05. Also see Supplementary Materials, Statistical Analyses.

We evaluated changes in the prefrontal neurophysiology associated with multielectrode recordings, which captures both neuronal ensembles (Fig S7) and local field potential (LFP) dynamics during IA discrimination. First, we recorded a 25-min baseline of activity in rPL on training day 1 during the IA discrimination task, and then trained rats in the IA discrimination task which involved placing them in the bright compartment of the aversive context and allowing them to freely explore the chamber (Fig. 4, A–B). Upon entry into the darkened compartment, rats received a single footshock, which resulted in increased rPL neuronal activity. Twelve of 29 rPL neurons (41%) displayed significant modulation, followed by increased activity thereafter (Fig. 4C). Footshock was also associated with modulated rPL LFPs across frequency bands (Fig 4D).

On testing day 3, we recorded neuronal activity in rPL during a 10-min retention test using the neutral context. Rats were returned to their home cage and allowed a rest period of 5 min. We then resumed recording during a 10-min retention test in the aversive context, and we compared neuronal activity changes between the aversive and neutral contexts.

To facilitate comparisons of rPL neuronal activity, we used principal component (PC) analysis, a data-driven approach that has been used widely to describe multivariate neuronal ensemble datasets.^84, 85^. Based on ensemble responses during exposure to the neutral context on testing day 3, we identified three components (PC1, PC2, and PC3) that accounted for 22%, 19%, and 15% of variance in neuronal activity, respectively (Fig. 4, E–F). Of these, PC3 was significantly increased in the neutral context relative to the aversive context (*p* < 0.05) (Fig. 4G). PC3 also exhibited a pre-latency increase, and a post-latency decline and recovery that parallels calcium transient activity in avBST during exposure to the neutral context on testing day 3 (i.e., Fig. 3J). By contrast, no differences were noted for the coefficient values of PC1 or PC2 as a function of either novel or training contexts (Fig. 4G), indicating that these PCs remained consistent during testing regardless of which IA context rats were exposed to. These data indicate that the shift in PC3 is associated with the rat’s experience in the neutral context.

Examination of rPL LFPs in the neutral and aversive contexts (Fig, 4, H–I) revealed increased low-frequency power in delta (1–4 Hz) and theta (4–8 Hz) bands during the 2-s post-transition epoch in the aversive context as compared with the post-transition epoch in the neutral context (Fig 4, I–K). Fascinatingly, during exposure to the aversive context both bands also showed an increase in power in the post-transition relative to the pre-transition epochs (Fig. 4, J–K). These data align with previous studies indicating that prefrontal activity in these frequency bands are associated with both cognitive and aversive processing.^86–89^ Taken together, these data suggest a role for rPL neuronal population activity and oscillatory power between safe and threatening contexts after IA discrimination learning.

### rPL–avBST excitation shifts memory consolidation toward improved fear discrimination.

**Figure 5.**
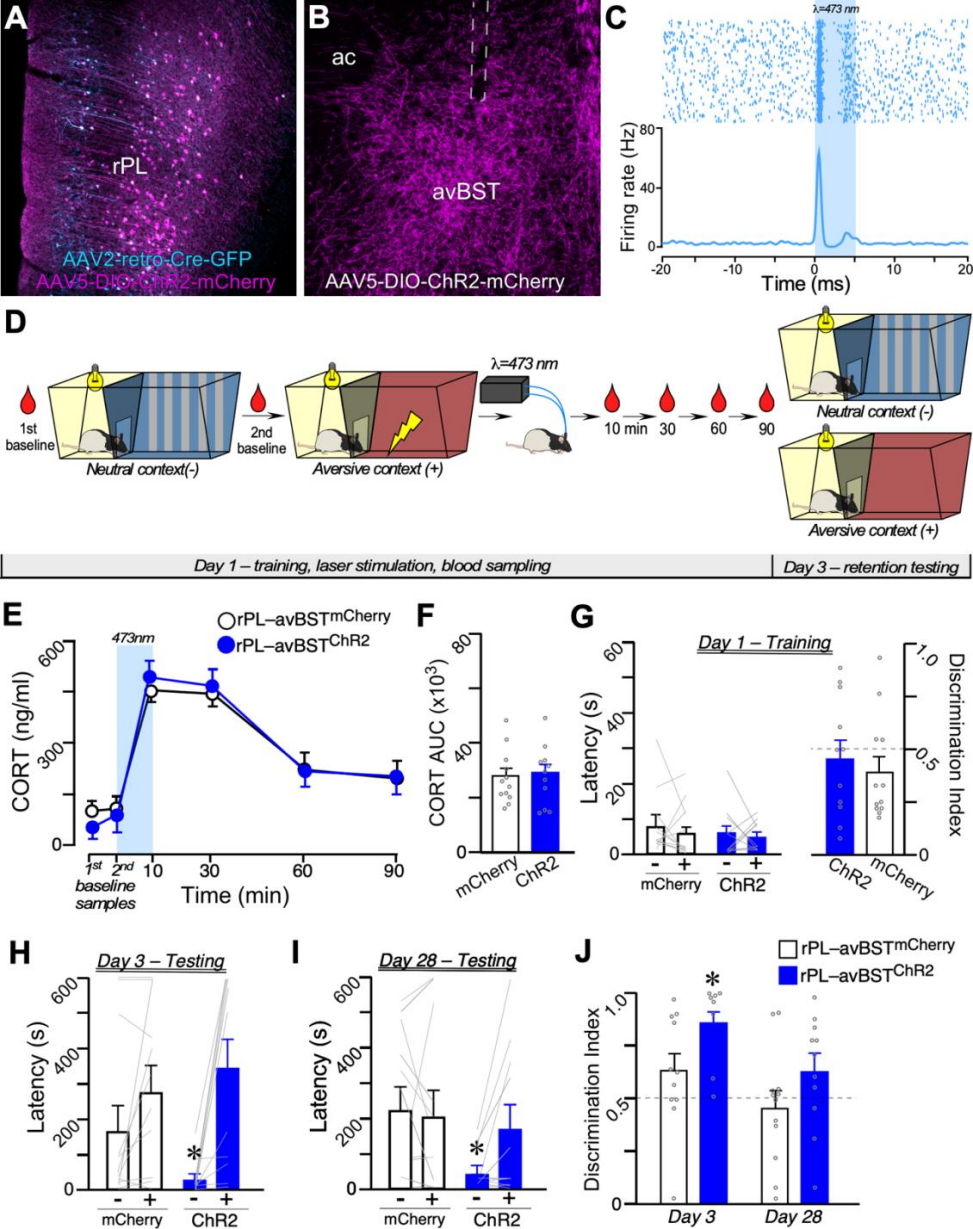
Post-training photoexcitation of rPL–avBST shifts memory consolidation toward improved fear discrimination. (**A–B**) Confocal images showing mCHerry fluorescent reporter expression in the rPL (**A**) and avBST (**B**) after making injections of AAV2-retro-Cre-GFP in the avBST and AAV5-DIO-ChR2-mCherry in rPL (GFP, cyan; mCherry, magenta). Dashed line, site of optical fiber placement. (**C**) Electrophysiological verification of ChR2 opsin functionality in the avBST. 473-nm laser stimulation (20-Hz, 5-ms pulse width) of axonal input from rPL evoked short-latency increases in the avBST neurons. (**D**) Illustration of behavioral procedures for the IA generalization task involving exposure to neutral and aversive contexts combined with repeated blood sampling and optical manipulation on training day 1. Red droplets indicate time points of blood collection for CORT assay. (**E**) Plasma CORT and (**F**) AUC values were not significantly different in rPL–avBST^mCherry^ and rPL–avBST^ChR2^ groups, indicating that rPL–avBST photoexcitation with ChR2 was ineffective at modulating stress hormonal activation. (**G**) On training day 1, the latencies to neutral (−) and aversive (+) contexts (*left*) and discrimination indices (*right*) are each low, as expected. (**H**) On testing day 3, latencies to the neutral and aversive contexts were comparably high for rPL–avBST^mCherry^ rats, whereas rPL–avBST^ChR2^ rats showed a significant reduction in the neutral (−) compared to aversive context. (**I**) On day 28 after training, the same groups of rPL–avBST^ChR2^ rats again demonstrated reduced latencies for the neutral (−) context relative to rPL–avBST^mCherry^ rats. (**J**) rPL–avBST^ChR2^ rats showed significantly better discrimination indices on day 3 relative to rPL–avBST^mCherry^ controls, and non-significant trending increased at day 28. n = 12, rPL–avBST^mCherry^; n = 11, rPL–avBST^ChR2^. *, *p* < 0.05 relative to rPL–avBST^mCherry^. Also see Supplementary Materials, Statistical Analyses.

The fact that rPL–avBST inactivation after IA training alters consolidation processes to increase fear generalization in adrenalectomized rats, suggests a more strictly mnemonic role for this pathway in memory modulation vis-à-vis exaggerated adrenocortical activity. Since these results indicate a necessity for rPL–avBST activity for preventing fear generalization, we next examined whether pathway excitation enhances fear discrimination. Since rats in the IA discrimination task successfully discriminate between the aversive and neutral contexts at baseline, we implemented a variation that promotes context generalization at baseline.^51, 72–74^ In the IA generalization task, rats were first allowed to freely explore the neutral context for 1 min on training day 1, but they did not receive a footshock when they entered into the darkened compartment. Rats were then transferred to the bright compartment of the aversive context and received a footshock 20 s after entering the darkened compartment. To further facilitate generalization, we increased footshock duration on day 1 of training in the aversive context (see Materials and Methods).^1, 78^ On testing day 3, when rats are exposed to neutral and aversive contexts, they displayed high retention latencies to each, which is indicative of generalization (e.g., Fig. 5H).

To activation the rPL–avBST pathway, rats received dual microinjections of AAV2-retro-Cre into the avBST bilaterally, and either a Cre-inducible AAV with the light-sensitive cation channel, channelrhodopsin-2, fused with mCherry,^90^ or a Cre-inducible AAV with mCherry alone in the rPL, followed by bilateral optical fiber implants that were positioned above the avBST (Fig. 5, A–B). A subset of ChR2-transduced rats was implanted with an optrode in the avBST to verify opsin efficacy in subsequent circuit manipulations. We measured optically-evoked excitatory neuronal responses in the avBST with 473-nm laser light for 10 min using stimulation parameters (20 Hz, 5-ms pulse width) that we previously established promotes maximal avBST neuronal activation.^44^ Excitation of rPL axonal input to the avBST with ChR2 consistently evoked excitatory short latency neuronal responses that were observed within 5 ms after each light pulse (Fig. 5C), supporting anatomical data suggesting that rPL–BST connections are monosynaptic (Fig. S8).

To assess adrenocortical output, rats were implanted with indwelling jugular catheters 2 d prior to training in the IA generalization task, to enable repeated blood sampling of CORT. During training (day 1), we collected a baseline blood sample, placed rats in the neutral context, collected a second sample, and transferred rats to the aversive context (Fig. 5D). Immediately after footshock, rats were returned to their home cages and then received 473-nm laser stimulation for 10 min. Repeated blood sampling ensued following laser illumination (i.e., 10 min post-footshock), and at 30-, 60-, and 90-min intervals after the footshock. On testing day 3, we assessed rats’ retention latencies for the neutral context followed by the aversive context (Fig. 5D).

During training on day 1, rats in both groups readily entered the darkened compartment of both neutral and aversive contexts. We did not observe any effects on adrenocortical output following post-training excitation in rPL–avBST^ChR2^ rats (Fig. 5, E–F), in contrast to the rPL–avBST circuit inhibition effect of exaggerating post-training increases in CORT (i.e., Fig. 2G). Upon testing day 3, rPL–avBST^mCherry^ rats exhibited similarly high retention latencies to both neutral and aversive contexts (Fig. 5H). Strikingly, the rPL–avBST^ChR2^ rats maintained low retention in the neutral context, as during training, although they displayed significantly higher levels of retention in the aversive context that were comparable to those of rPL-avBST^mCherry^ rats (Fig 5H). Prior work suggests that contextual memories show greater generalization when tested weeks later.^91, 92^ To assess the persistence of enhanced memory specificity indicated by the rPL–avBST^ChR2^ group on testing day 3, we retested rats on day 28. This testing displayed that rPL–BST^ChR2^ rats displayed a similar pattern, albeit to a lesser degree, showing higher latencies to the neutral context versus the aversive context, whereas the rPL–avBST^mCherry^ group still maintained comparably high levels of retention in each context (Fig. 5I). Further, rPL–avBST^ChR2^ rats exhibited higher discrimination indices on day 3 than rPL–avBST^mCherry^ controls, although these values were not significantly on day 28 (*p* = 0.1) (Fig. 5J). In follow up, we repeated the same experiment, instead using an intersectional chemogenetic strategy to activate the rPL–avBST circuit post-training, with injections of clozapine-*N*-oxide (CNO; 1 mg/kg, IP) in rPL–avBST^mCherry^ and rPL–avBST^hM3Dq^ groups (Fig. S9). As with optogenetic excitation, post-training chemogenetic activation of the rPL–avBST pathway altered consolidation of fear discrimination between the neutral and aversive contexts, without influencing overall memory strength for the training context itself.

We investigated the specificity of post-training manipulations of the rPL–avBST circuit on memory specificity by targeting an additional projection stemming from the rPL, the ventrolateral (vl) PAG, because evidence indicates that there are collateralizations to the vlPAG from axons in the rPL–avBST pathway.^93^ Moreover, a previous report showed that a prefrontal–vlPAG circuit controls the expression of contextual fear discrimination in mice.^22^ Using a combination of viral tracing approaches, we confirmed that avBST-projecting rPL neurons collateralize extensively to vlPAG (Fig. S10). Next, we functionally evaluated the rPL–vlPAG pathway in IA discrimination, using the IA generalization task to bias baseline behavioral responses toward fear generalization. We tested rats containing injections of AAV with ChR2 in the rPL, under the control of the CaMKIIα promoter, and optical fiber implants above the vlPAG. Controls received the same procedures, except were injected with AAV that expressed YFP instead of ChR2 (Fig. S10). On training day 1, after exposing rats to neutral and aversive contexts, we performed 473-nm laser light excitation (20 Hz, 5-ms pulse width) for 10 min. On testing day 3, rats in the rPL–vlPAG^YFP^ control and rPL–vlPAG^ChR2^ groups displayed comparable levels of generalization to both the neutral and training contexts (Fig. S10). In summary, activity in the rPL–vlPAG pathway is not involved in the early post-training consolidation period and provides convergent and consistent evidence supporting the rPL–avBST pathway as a modulator of memory specificity.

## DISCUSSION

We characterized neural processing in the rPL and avBST during the immediate aftermath of an aversive event, using a combination of anatomy, endocrinology, fiber photometry, neurophysiology, optogenetics, and chemogenetics. Our results indicate that the rPL–avBST pathway is both necessary and sufficient for modulating the consolidation of fear generalization independently of increases in adrenocortical activation. These findings illustrate a novel contribution of the rPL–avBST pathway in long-term behavioral expression of memory specificity and may provide insight into how circuit dysregulation may underlie fear over-generalization in stress-related psychiatric illnesses.

### mPFC circuitry and fear discrimination

Prior work examining mPFC circuitry in aversive processing found that it plays an important role in fear learning.^22, 24, 34, 94, 95^ However, these studies have generally focused on mPFC circuitry in fear expression versus consolidation as addressed in the present report. mPFC activity on its own is associated with the inhibition of retrieval to an auditory conditioned stimulus following Pavlovian learning,^24^ and may inhibit tone discrimination via interaction with somatostatin neurons in the basolateral amygdala.^96^ Evidence also indicates that rPL is associated with decreasing avoidance during extinction of fear learning,^53^ an effect consistent with work suggesting that rPL activation decreases avoidance during retention in a platform-mediated avoidance task.^52, 97^ Recruitment of PL neuronal ensembles during the transition between non-threatening and threatening contexts is important to distinguish between environments,^98, 99^ whereas during retrieval of contextual fear memories, activation of a subset of the vlPAG-projecting neuronal subpopulation is necessary and sufficient to promote changes in memory specificity.^22^ The IL projection to BST has also been implicated in modulating the retrieval of contextual fear in settings when conditioned and unconditioned stimulus pairings may be less well associated.^34, 35^

Our results are consistent with prior studies implicating mPFC in discriminatory aspects of fear memory expression and retrieval. However, the present findings provide a new conceptualization that involves rPL–avBST circuit activity changes after an aversive experience that in turn modulates the mnemonic properties of the accompanying contextual features. Our in vivo recording data identified rPL neuronal ensemble changes in response to footshock, and at retention testing they identified rPL activity and LFPs that were distinct between the neutral context relative to the aversive context. We report changes in frequency bands in rPL that are reminiscent of previous studies, whereby increases in theta power indicate a recognition of the aversive context that was previously associated with a footshock.^24, 88, 100^ Moreover, post-training optogenetic excitation and inhibition of rPL–avBST pathway enhanced and attenuated memory specificity, respectively. Of relevance to the work of Rozeske and colleagues,^22^ we noted collateralization between rPL projections to both the avBST and vlPAG (Fig. S10). Nevertheless, our optogenetic targeting of the rPL–vlPAG pathway immediately following IA training did not influence future generalization. While we did not explicitly examine IA memory expression, our results indicate that increasing rPL activity is associated with encoding, consolidation, and expression. This raises the prospect that mobilization of distinct pathways from rPL are important for different phases of learning, with the projection to the avBST being important for consolidation. However, it is unknown whether the rPL projection to the avBST modulates mnemonic processes beyond consolidation of memory specificity, or whether rPL modulation of acquisition and retrieval is regulated via other circuits.

### BST role in fear memory

Many studies have observed BST involvement in aversive learning, including IA training,^48–50, 101, 102^ and contextual and auditory fear conditioning.^35, 36, 103–105^ Nevertheless, BST is also implicated in aversive behavioral changes situationally, such as during stress coping,^44, 54, 106^ and in settings that favor anxiety-like responses.^107–110^ Work in recent years has highlighted the extensive heterogeneity of BST functions, which appear to largely depend on subregion or a neurochemical phenotype.^111, 112^ For instance, activation of dorsal aspects of BST tend to evoke anxiety-related behavioral changes,^108–110, 113^ or aversion,^114^ whereas ventral and posterior regions have been implicated in regulating behavioral avoidance and neuroendocrine activation.^43, 44, 54, 115^

Our results suggest that avBST activity changes during fear discrimination are not directly associated with avoidance, but instead, approach behavior in the IA chamber. During IA training, activity decreases in avBST were observed after rats made the association between the aversive context and footshock, whereas during retention testing avBST activity increased in the interval immediately preceding the transition from the brightly lit to the darkened compartment of the neutral context. This increase occurred regardless of rats’ individual latencies to make this transition. That we observed minimal changes in avBST activity prior to general exploratory transitions, in a context distinct from the shock-context association altogether (i.e., Fig. S6), lends further support that avBST mediates approach in stress-inducing contexts, as has been observed elsewhere.^83^ Taken from the perspective that BST mediates uncertainty or integrates information related to negative valence,^36, 116^ our results indicate a novel role for avBST activity following an aversive event to promote long-lasting changes in memory specificity.

### Glucocorticoids and memory consolidation

One major question raised by our data has to do with the relationship between endogenous changes in adrenocortical activity and the consolidation of memory strength and specificity. Extensive evidence has shown that exogenously increasing glucocorticoids is sufficient to enhance memory consolidation for emotionally arousing stimuli (for further review see ^11, 117^). Post-training administration of CORT or the synthetic glucocorticoid, dexamethasone, enhances long-term memory for training experiences that are emotionally arousing, including inhibitory avoidance,^118–120^ contextual and auditory fear conditioning,^121^ as well as spatial and novel-object recognition.^122^ During the post-training period CORT has been reported to predominantly activate the Type II glucocorticoid receptor (GR) within the basolateral amygdala, hippocampus, and mPFC,^123, 124^ as local administration of a selective Type II GR agonist into any of these regions enhances memory consolidation for many types of stressful experiences.^125, 126^ Subsequent work extends the role of glucocorticoids to promoting memory generalization.^2, 75^ Post-training systemic CORT administration enhances generalization to a novel environment,^1, 75–77^ or cue,^78^ and this is associated with alterations in amygdala and hippocampal activity.^78^

Our observation that post-training administration of CORT in rats enhanced generalization to a neutral context is consistent with the interpretation that glucocorticoids are sufficient to enhance generalization. Nevertheless, past work has established that rPL, avBST, or rPL–avBST inactivation each lead to augmented HPA activation and CORT output.^44, 45, 54, 127, 128^ That we observed augmented CORT levels following post-training inhibition of the rPL–avBST pathway (i.e., Fig. 2G) led us to hypothesize that increased levels of CORT may contribute to the enhancement of generalization. Our previous work further indicates that the endogenous increase in CORT resulting from post-training inactivation of the avBST in IA learning is necessary to enhance fear memory strength, although we did not test generalization in that study.^47^ However, to our surprise, when we prevented stress-induced elevations in CORT in rats with their adrenal glands removed, this failed to prevent generalization produced by post-training inactivation of the rPL–avBST pathway.

Thus, augmented CORT following rPL–avBST inactivation does not appear to contribute significantly to context generalization. This potential mismatch for how circuit-mediated increases in CORT could enhance memory strength in one setting (avBST inhibition, e.g., see ^47^), but not memory generalization in another (rPL–avBST inhibition, this study), raises several scenarios for future study. The first is that the avBST is responsible for mediating glucocorticoid-dependent effects on memory consolidation, and/or is mediated via another upstream region aside from rPL. A second possibility is that activation of additional excitatory input converging onto the avBST (e.g., ventral subiculum, basomedial amygdala ^46, 106^) may be necessary to drive the glucocorticoid-mediated enhancement of memory consolidation. Finally, it will be important to establish whether the avBST, or its projection neurons in upstream regions, serve as key sites of action for GR-mediated effects on aspects of memory consolidation.

### Functional implications

Prior knowledge can help determine the degree of threat in subsequent experiences. This is a fundamental aspect of the nervous system that enables adaptation under a variety of environmental conditions. The behavioral consequences of memory specificity necessarily involve the comparison of current experiences with past experiences, and thus implicate retrieval-related processes in altering memory specificity. Nevertheless, the current study directs attention to a novel function for a circuit and process, whereby activity changes in the immediate aftermath of an aversive event modulate consolidation of memory specificity, which subsequently has a long-lasting impact on behavior. Moreover, the current study offers new insight about a network that is important for the consolidation of features that are unrelated to memory strength. While it is widely recognized that salient experiences concurrently enhance both memory strength and generalization, these results direct attention to the prospect that they are dissociable under some circumstances.

The functional relevance regarding rPL–avBST modulation of the HPA axis in the aftermath of an aversive experience is unclear. Other brain regions and circuits, particularly within the limbic forebrain, have been shown to modulate HPA activation in response to threats from the environment.^46^ Whereas the rPL–avBST pathway is not unique in this regard, the glucocorticoid independence of its effect on memory consolidation in the aftermath of a stressor suggests another functional role, most likely that of priming the organism to subsequent inflammatory or injury-related stimuli that likely ensue under such adverse conditions.^129, 130^ Thus, environmental conditions that disfavor prefrontal engagement—such as in exigent life-threatening circumstances or following chronic stress exposure—would promote fear memory generalization and the ensuing neuroendocrine consequences, which optimize behavioral and physiological adaptation in tandem.

Elucidating neural responses to traumatic experiences has importance to human diseases. Increased threat generalization in humans is a feature of anxiety and post-traumatic stress disorders.^131–134^ Improved threat-safety discrimination is an important classifier in human studies on resilience,^133, 135^ and recent evidence suggests the same may be true in rodents.^136^ Our results point to the possibility that rPL–avBST circuit activation after a traumatic event promotes resilience by limiting the degree of generalization and glucocorticoid activation, and that a diminution in this pathway shifts the individual toward susceptibility.

## Supplementary Material

Supplement 1
