# Supplementary material for: Prefrontal projections to the bed nuclei of the stria terminalis modulate the specificity of aversive memories
Source: Res Sq. 2024 Oct 21:rs.3.rs-4241372. Preprint. [Version 1] doi: 10.21203/rs.3.rs-4241372/v1 (PMC11577250; doi:10.21203/rs.3.rs-4241372/v1)
Supplement: Supplement 1 [file NIHPPrs4241372v1-supplement-1.pdf]

SUPPLEMENTARY MATERIALS

This section includes:

- *-Supplementary Figures – 10 figures*
- *-Materials and Methods – complete description of methods*
- *-Statistical Analysis – delineation of all statistical information in table format*

SUPPLEMENTARY FIGURES

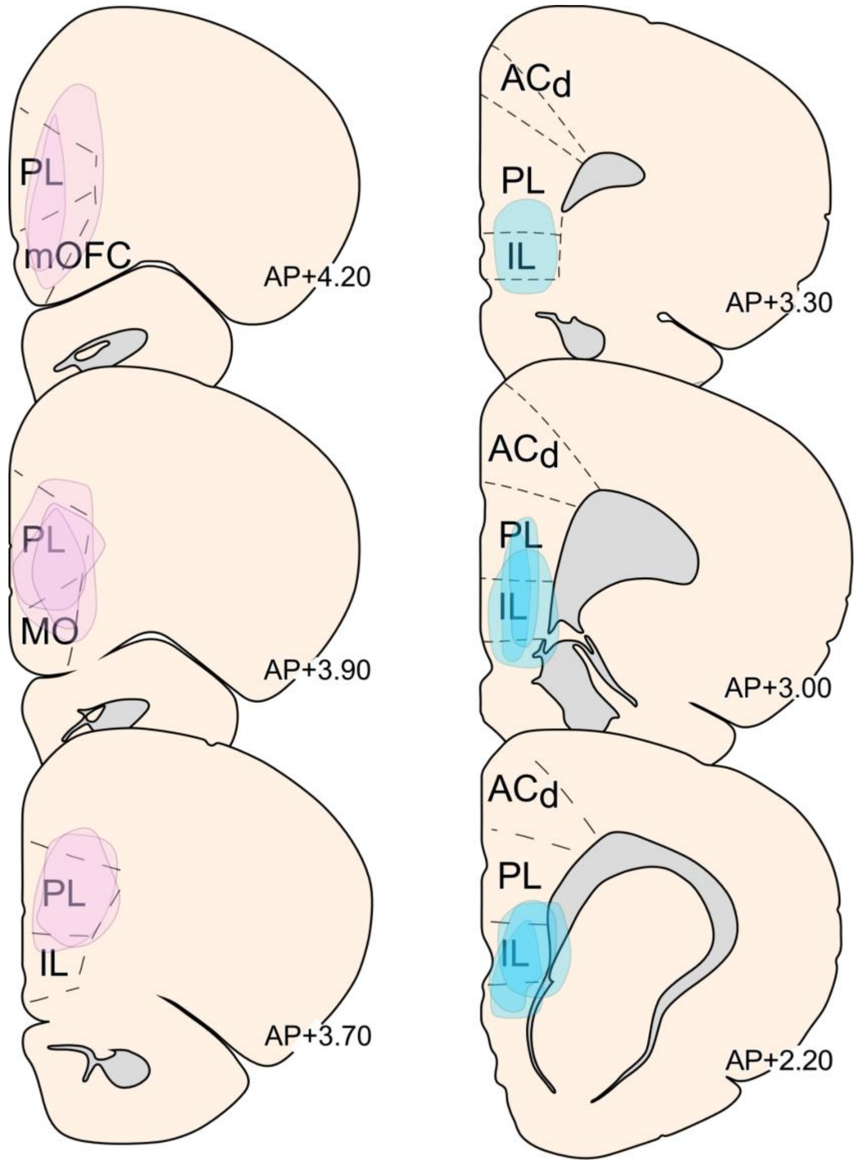

Figure S1. Mapping of microinjections in prefrontal subfields. *Left*, Illustration of the anterior-to-posterior extent of viral expression (in magenta) following injection of AAV5-CAMKIIα-mCherry in the rPL subfield (n = 3). *Right*, Illustration of the antero-posterior extent of viral expression (in cyan) following injection of AAV5-CAMKIIα-GFP in IL (n = 3).

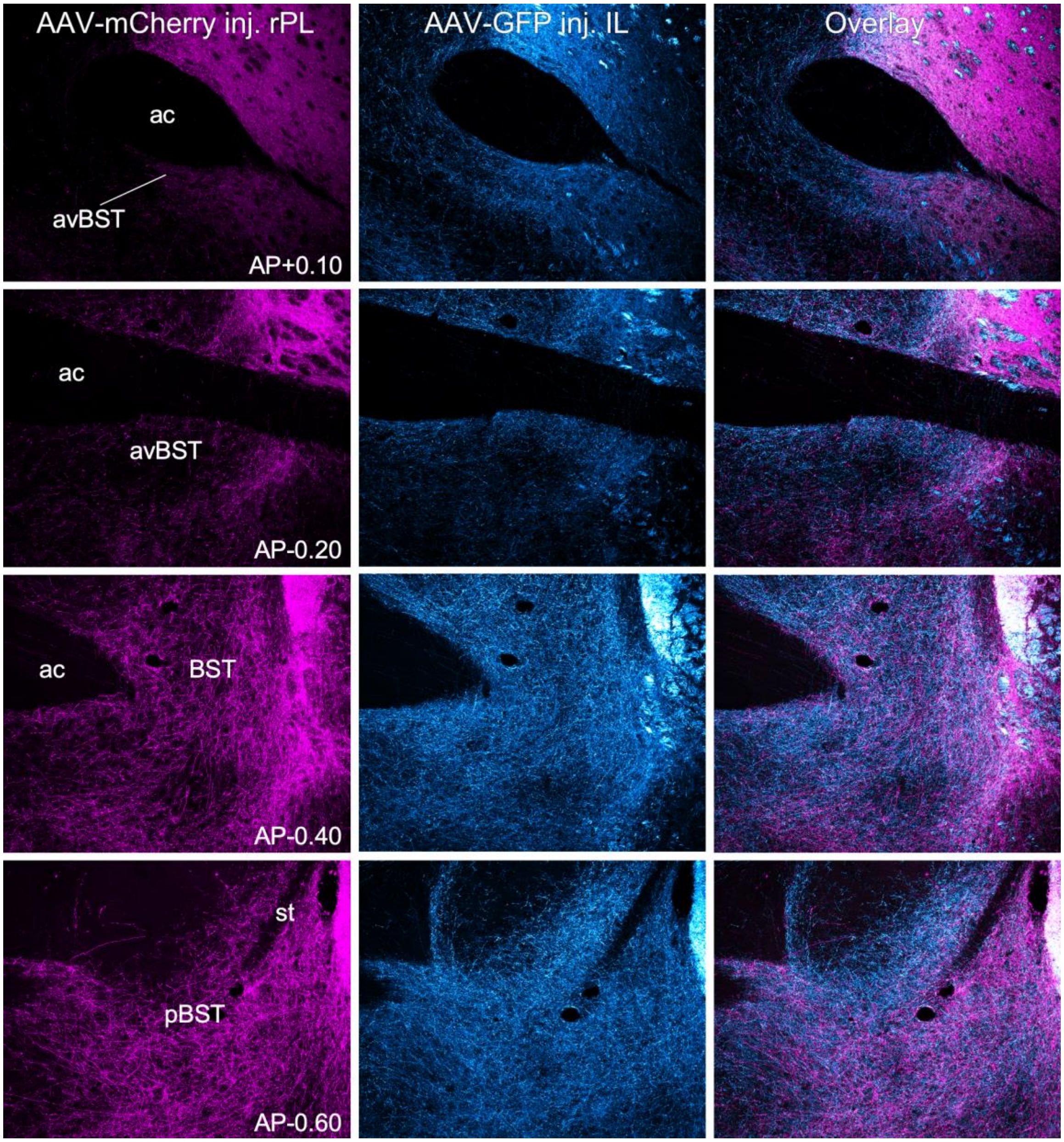

Figure S2. Characterization of rPL and IL terminal fields across the anteroposterior extent of BST. Confocal series of coronal sections in one rat that received dual viral injections in rPL and IL. *Left column*, AAV5-CAMKIIα-mCherry injected in the rPL exhibited sparse labeling of BST (magenta) at rostral levels that increased in density toward the caudal aspect, particularly within the avBST. *Middle column*, AAV5-CAMKII-GFP injected in IL exhibited dense labeling across BST (cyan). *Right column*, Overlay of GFP and mCherry prefrontal terminal labeling across BST.

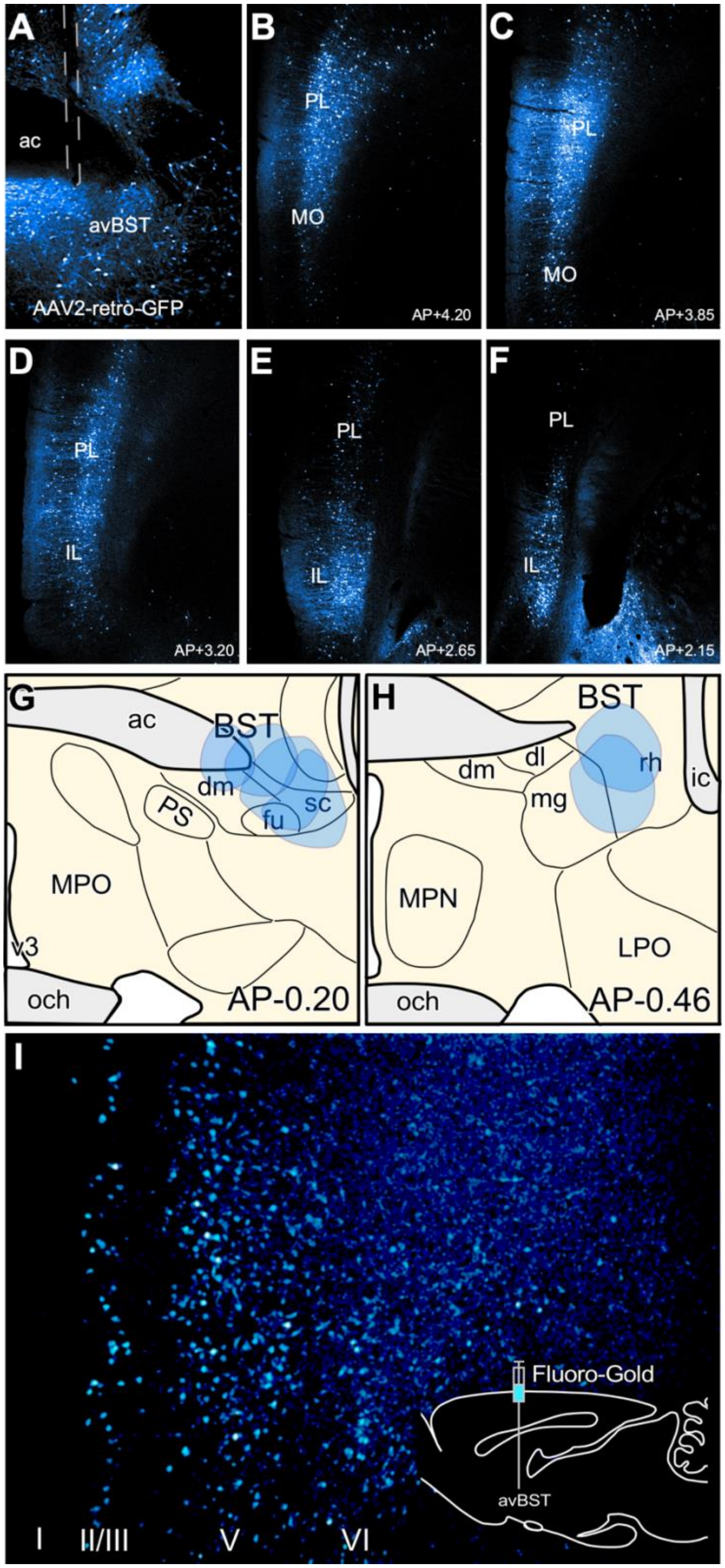

Figure S3. Retrograde transport techniques confirm an rPL projection to the avBST. (**A**) Representative photomicrograph depicting microinjection of retrogradely labeled

AAV2-retro-GFP (cyan) in the avBST. (**B–F**) Confocal images of retrograde expression of AAV2-retro-GFP throughout the antero-posterior axis of the mPFC reveal that projections to the avBST hail from more rostral aspects of PL. (**G, H**) Illustration of FG injection sites in the avBST (n = 6) shown over two representative distances relative to bregma. (**I)** Image in rPL showing an example of retrograde accumulation of FG tracer in rPL principal neurons. BST subdivisions: dl, dorsolateral; dm, dorsomedial; fu, fusiform; mg, magnocellular; rh, rhomboid; sc, subcommissural); ic, internal capsule; MPN, median preoptic nucleus; MPO, medial preoptic area; och, optic chiasm; PS, parastrial nucleus; v3, third ventricle.

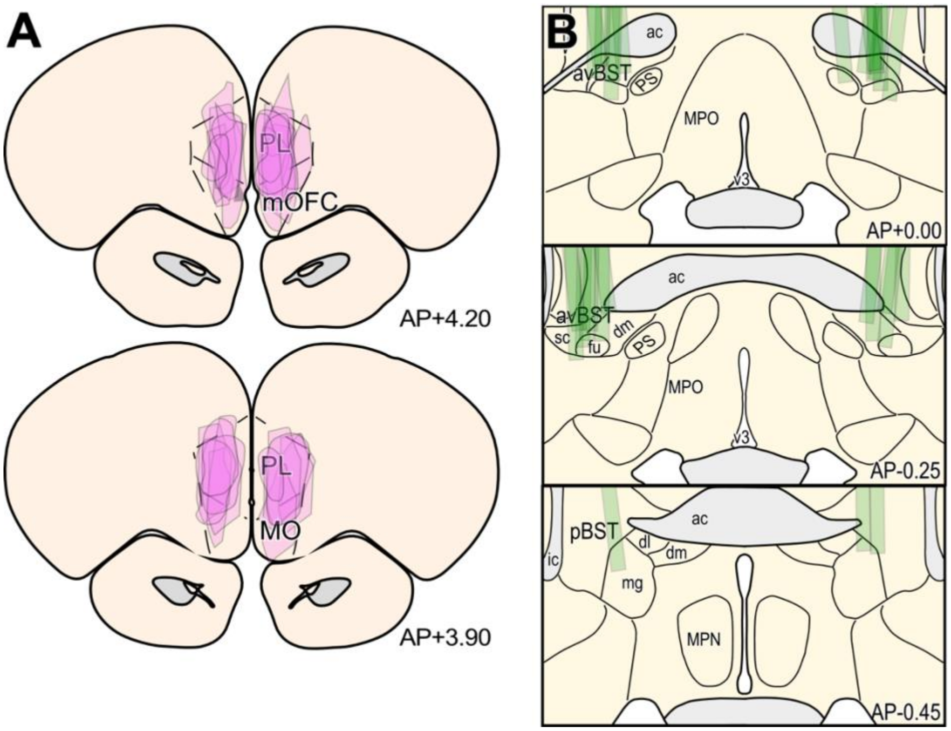

Figure S4. Viral and optic placements in the rPL and avBST. (**A**) Extent of fluorescent labeling of DIO-mCherry-expressing neurons in rPL after recombination with AAV2-retro-cre. (**B**) Illustration of optic placements in BST, from the rPL–avBST^{Halo} group (n = 15).

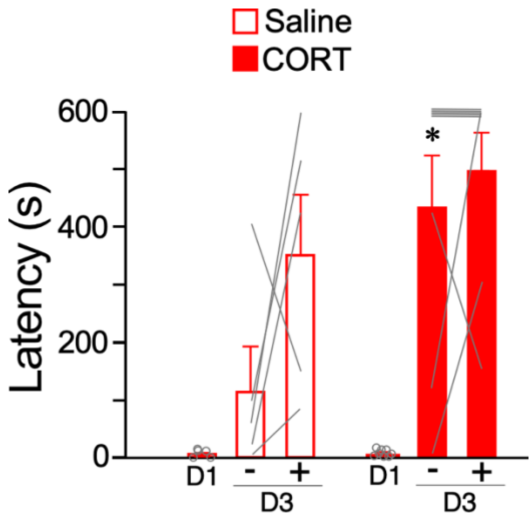

Figure S5. Post-training intraperitoneal injection of CORT increases latency for the neutral context 3 d after training. Rats were trained in the IA discrimination task. Latencies are shown in the aversive context on day 1 (D1), and on testing day 3 (D3), in neutral (-) and aversive (+) contexts. Injections of CORT (3 mg/kg, i.p.) given immediately after IA training in the aversive context on day 1, led to significant increase in the latency to avoid the neutral context relative to saline-injected rats on testing day 3. *, $p < 0.05$, latency increase in CORT relative to the saline group in the neutral context. n = 5, saline; n = 8, CORT. Also see *Supplementary Materials, Statistical Analyses*.

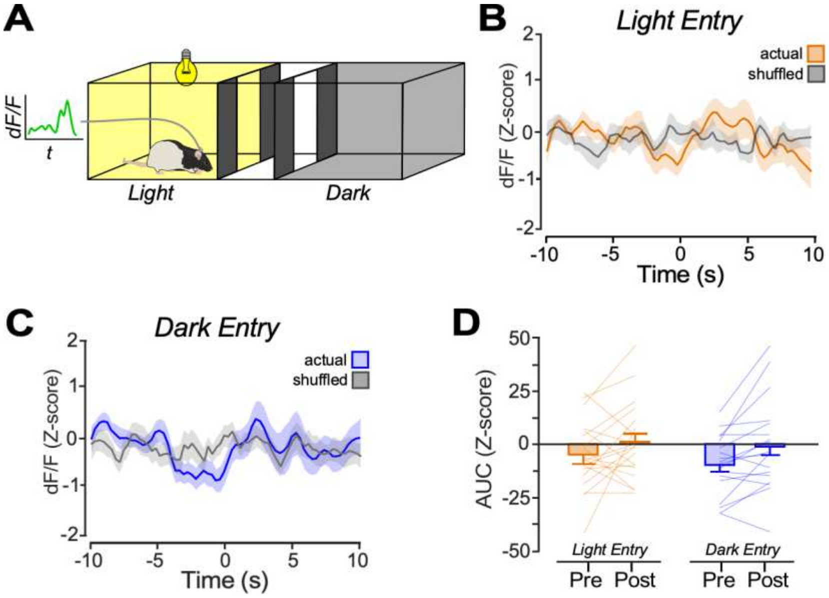

Figure S6. avBST calcium transient changes during context transitions using a three-chamber apparatus. (**A**) Illustration of behavioral and fiber photometry procedures. (**B**) Entries into the light compartment were logged and avBST GCaMP8s dF/F z-scored activity was aligned to time 0. Mean ± SEM overlaid on shuffled data randomly sampled from dF/F values across time. (**C**) Entries into the dark compartment were logged, and avBST activity was aligned to time 0. (**D**) AUC values pre (-5–0 sec), and post (0–5 sec) for light and dark entries. *, $p < 0.05$, relative to Dark Entry/Post. Also see *Supplementary Materials, Statistical Analyses*.

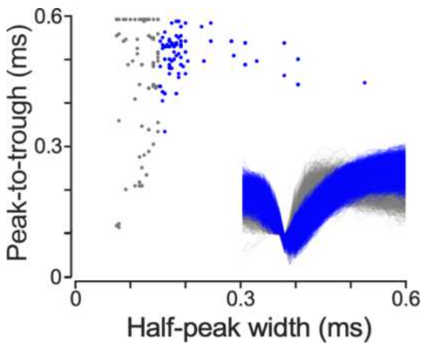

Figure S7. rPL neuronal waveform characteristics. Half-peak width (ms) and peak-to-trough duration (ms) were used to isolate excitatory neurons (blue circles). The inset displays example waveforms in blue for analyzed excitatory neurons.

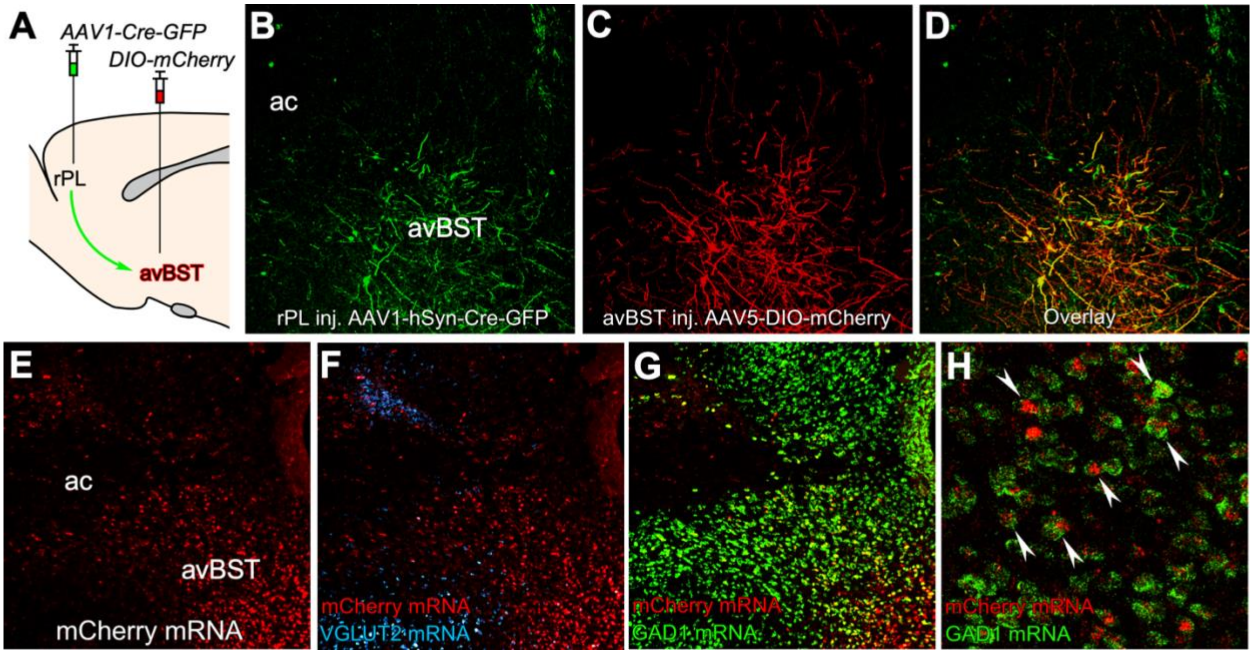

Figure S8. Anatomical evidence for rPL monosynaptic connections with GABAergic neurons in the avBST. (**A**) Midsagittal diagram illustrating viral approach for trans-neuronal tracing using microinjection of AAV1-cre-GFP in rPL and cre-dependent AAV5-DIO-mCherry in avBST. The red outline for "avBST" indicates expression of mCherry in the avBST neurons that receive transneuronal spread from rPL axon terminals (**B–D**) Representative confocal image of BST illustrates AAV1-hSyn-Cre-GFP expression (**B**), AAV5-DIO-mCherry (**C**), and overlay of fluorescent labeling (**D**) in the avBST. (**E–H**) Fluorescent in situ hybridization reveals an abundance of neurons postsynaptic to rPL that are GABAergic (**G**, **H**), but not glutamatergic (**F**), as based on cellular colocalization of viral mCherry and endogenous VGLUT2 GAD-1 mRNA transcripts.

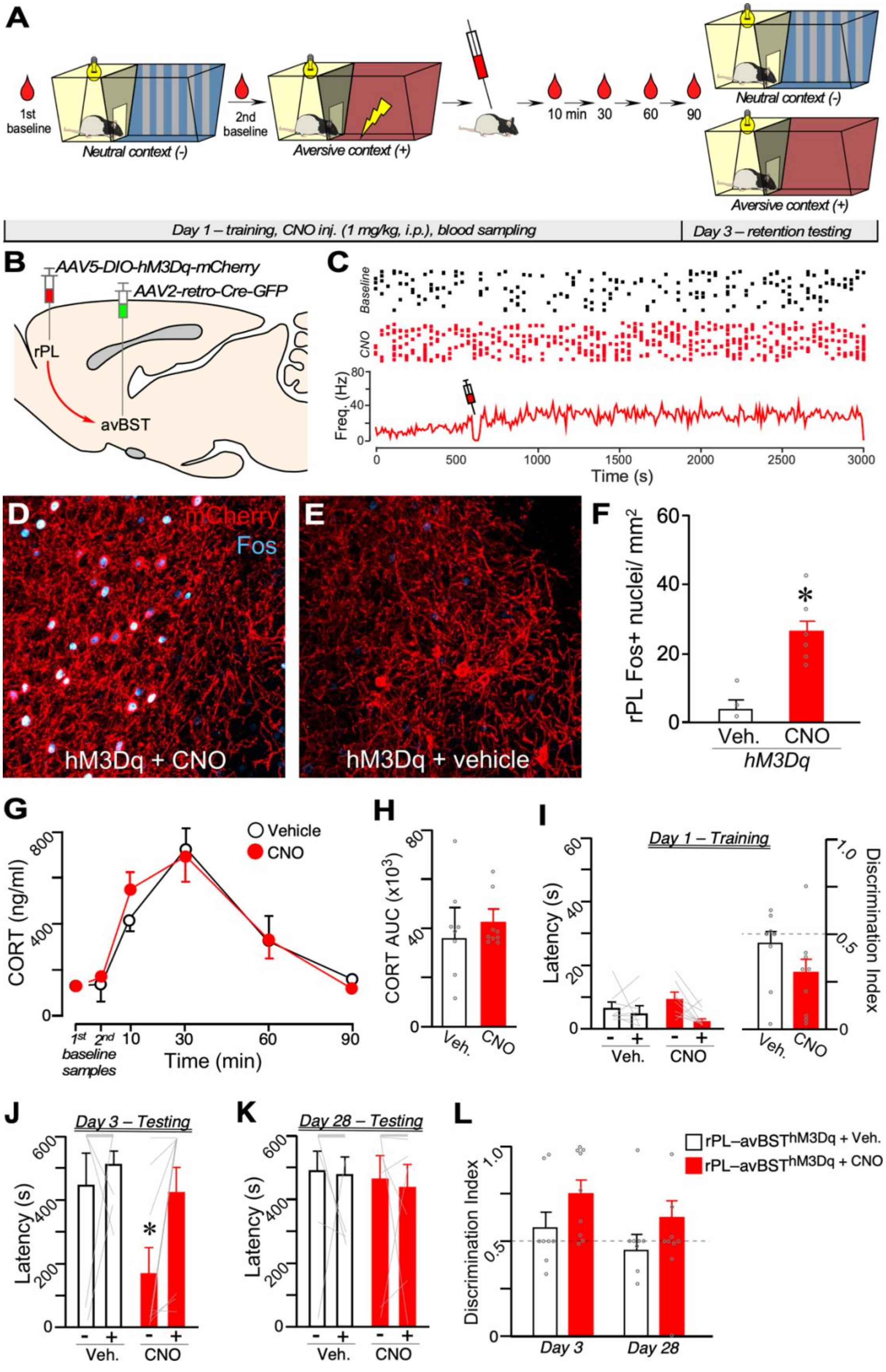
A
1st baseline
Neutral context (-)
2nd baseline
Aversive context (+)
10 min
30
60
90
Neutral context (-)
Aversive context (+)
Day 1 – training, CNO inj. (1 mg/kg, i.p.), blood sampling
Day 3 – retention testing
B
AAV5-DIO-hM3Dq-mCherry
AAV2-retro-Cre-GFP
rPL
avBST
C
Baseline
CNO
Freq. (Hz)
Time (s)
D
mCherry
Fos
hM3Dq + CNO
E
hM3Dq + vehicle
F
rPL Fos+ nuclei/ mm2
Veh.
CNO
hM3Dq
G
Vehicle
CNO
CORT (ng/ml)
1st 2nd baseline samples
Time (min)
H
CORT AUC (x10^3)
Veh.
CNO
I
Day 1 – Training
Latency (s)
Discrimination Index
J
Day 3 – Testing
Latency (s)
K
Day 28 – Testing
L
rPL–avBST^hM3Dq + Veh.
rPL–avBST^hM3Dq + CNO
Discrimination Index
Day 3
Day 28

Figure S9. Chemogenetic excitation of rPL–avBST pathway shifts memory consolidation toward improved fear discrimination. (**A**) Diagram of IA generalization task, post-training chemogenetic rPL–avBST pathway excitation, and repeated blood sampling for analysis of CORT. Rats received an injection of clozapine-*N*-oxide (CNO; 1 mg/kg, IP) immediately following training on day 1. (**B**) Illustration depicting viral strategy for chemogenetic targeting of the rPL–avBST pathway. (**C**) Electrophysiological recording, in vivo, in an anesthetized rat bearing viral expression in the rPL–avBST pathway, to verify that chemogenetic excitation (CNO, 1 mg/kg, IP) increases activity in avBST projector neurons in rPL. *Top*, Raster plot around randomly selected time points prior to (black) and following (red) injection of CNO for a single unit isolated. *Bottom*, Firing rate (Hz) for a single unit isolated in the avBST. (**D–E**) Confocal fluorescent image showing c-Fos immunolabeling in avBST-projector neurons in rPL. Fos immunolocalization is increased in mCherry expressing neurons following CNO injection (**D**) relative to saline vehicle-injected (**E**) rats. (**F**) Quantification of Fos-immunoreactive nuclei in rPL as a function of CNO treatment in rats bearing hM3Dq expression in the rPL–avBST pathway. (**G–H**) Radioimmunoassay for plasma CORT prior to and following training on day 1 revealed no differences between rPL–avBST$^{hm3dq\text{-}CNO}$ and rPL–avBST$^{hm3dq\text{-}Veh}$ rats, at any time points or in the AUC. (**I**) Latency values for training on day 1, and discrimination indices for vehicle- and CNO-receiving rats. (**J**) Whereas rPL-avBST$^{hm3dq\text{-}Veh.}$ rats displayed high latency values for both aversive (+) and neutral (-) contexts, rPL–avBST$^{hm3dq\text{-}CNO}$ rats displayed significantly lower latencies for the neutral context (-) compared to the training context. (**K**) Latencies 28 d after training in the same rats did not show group differences, as both exhibited high latencies for each context. (**L**) Discrimination indices for testing on day 3 and day 28. *, $p < 0.05$, relative to rPL–avBST$^{hm3dq\text{-}Veh.}$ in the neutral (-) context. n = 8, rPL–avBST$^{hm3dq\text{-}Veh.}$; n = 9, rPL–avBST$^{hm3dq\text{-}CNO}$. Also see *Supplementary Materials, Statistical Analyses*.

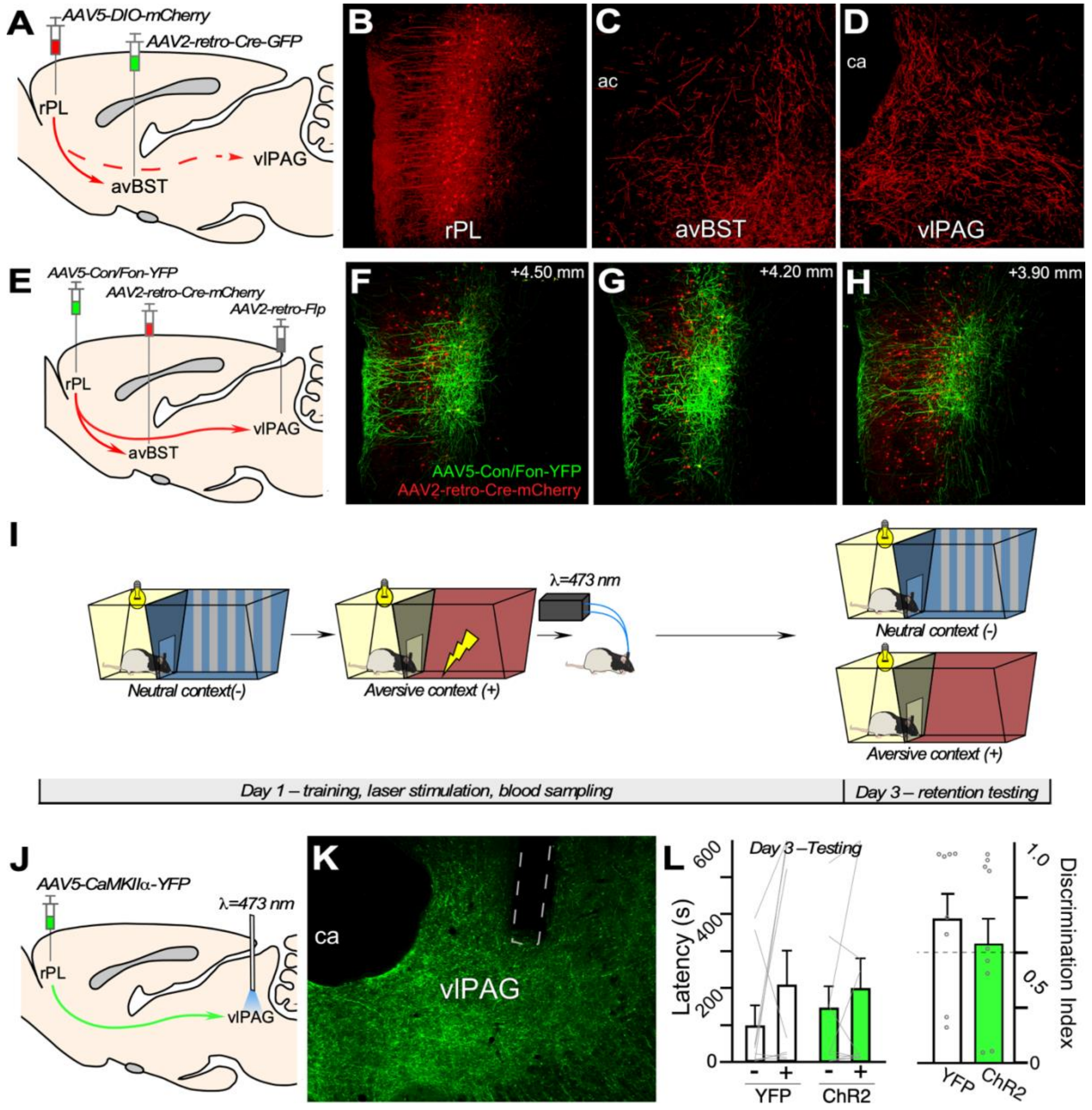

Figure S10. Post-training rPL–vlPAG pathway excitation does not alter memory specificity. (**A**) Viral strategy for characterizing collateralization between the rPL–avBST and rPL–vlPAG pathways. (**B–C**) Cre-dependent mCherry labeling of avBST-projector neurons in the rPL and avBST. (**D**) avBST projector neurons in rPL also display axon collateralization in the vlPAG. (**E**) Intersectional viral approach for verification of rPL axonal collateralization to avBST and vlPAG. (**F–H**) Confocal images showing Con/Fon-yellow fluorescent protein (YFP) expression at various anterior-to-posterior aspects of rPL. YFP expression depends on recombination with both retrogradely transported AAV2-retro-Cre-mCherry from avBST and AAV2-retro-Flpo from vlPAG. (**I**) Behavioral strategy for IA generalization task with post-training optogenetic excitation of the rPL–vlPAG pathway. (**J**) Viral strategy for optogenetic excitation of rPL axonal projections in vlPAG. (**K**) Representative fluorescent image of axonal innervation of vlPAG from rPL and optical

fiber placement (dashed line). (**L**) On testing day 3, latency values for rPL–vlPAG^{YFP} and rPL–vlPAG^{ChR2} groups are similar during exposure to both neutral (-) and aversive (+) contexts. *Right*, Discrimination indices were also similar between groups. n = 9, rPL–vlPAG^{YFP}; n = 9, rPL-vlPAG^{ChR2}. Also see *Supplementary Materials, Statistical Analyses*.

MATERIALS AND METHODS

Subjects. Adult male Long-Evans or Sprague-Dawley rats, 225–250 g at time of arrival (Charles River Laboratories), were used for all experiments. Upon arrival, rats were acclimated for at least 7 d prior to surgery in an AAALAC-approved vivarium with ad libitum access to food and water. All procedures were approved by the University of Iowa Office of the Institutional Animal Care and Use Committee and in accordance with the National Institutes of Health Guide for the Care and Use of Laboratory Animals.

Surgeries. Optogenetic/fiber photometry experiments. Rats were anesthetized with 4% isoflurane in oxygen and were placed in a stereotaxic frame (Kopf Instruments), in which they received a presurgical analgesic (2 mg/kg Meloxicam, subcutaneous [SC]). Surgical anesthesia was maintained at 1.5%–2.0% isoflurane. Rats received bilateral microinjections (350 nL per side) of AAV solution directed at the avBST (anteroposterior, AP: -0.18 mm relative to bregma; mediolateral, ML: ±1.20 mm; dorsoventral, DV: -6.75 mm) and the rPL region (AP: 3.85 mm; ML: 0.85 mm; DV: 3.00 mm). Following AAV injection, steel ferrules (Plastics One) fitted with fiber optics (200 µm diameter, 0.37 NA; Thorlabs) for optogenetic experiments were placed bilaterally immediately dorsal to the avBST (AP: -0.18mm; ML: 2.35 mm; DV: -6.55 mm; 6°) and then secured with dental cement and surgical screws. Fiber photometry experiments involved unilateral AAV injections and optic placements directed at the same rPL or avBST coordinates. Rats were allowed to recover for at least 5 w and then were habituated to a holding room, where blood collection was handled by an experimenter daily, for 1 w prior to behavioral procedures.

Hormone assays. Two days prior to IA training, rats were implanted with indwelling jugular catheters as described elsewhere.[62, 137] Under isoflurane anesthesia, polyethylene (PE-50) tubing containing sterile heparin saline (50 U/mL) was implanted with its internal SILASTIC (Dow Corning) tip positioned at the atrium and the remaining length exteriorized at the nape in the interscapular region. On the experiment day, and at the beginning of the circadian trough (AM, 0600), rats were brought to a procedure room where the jugular catheters were connected to 1-mL syringes containing sterile heparin saline. Following at least 90 min to allow for habituation, blood samples (~200 µL) were taken prior to IA training (0 min) for baseline estimation of CORT levels. Subsequent samples were taken immediately following IA training at repeated intervals (10, 30, 60, and 90 min). Each sample was immediately place in a chilled 1.5-mL microcentrifuge tube containing 15 µL EDTA/aprotinin and centrifuged for 20 min prior to plasma fractionation and storage at -80°C. Plasma CORT was measured without extraction with rabbit antisera raised against CORT-BSA with ^{125}I-CORT-BSA as a tracer (MP Biomedicals). Intra- and inter-assay coefficients of variation were 5% and 10%, respectively, with a sensitivity of 8 ng/mL.

Behavioral procedures. All rats were trained on a single-trial step-through inhibitory IA task,[79, 138] with modified procedures to assess discrimination and generalization, as based on.[51, 72] Multiple IA apparatuses were used, each of a similar general design, albeit with distinct contextual modifications. General features of the IA apparatus were a trough-shaped box segmented into two compartments: one with an illuminated white

plastic bottom and the other with a darkened stainless-steel bottom. The darkened stainless-steel portion of the apparatus was connected to a shock generator and timer. A retractable stainless-steel door separated the two compartments. IA chambers were made distinct with the following features: (1) Aversive context: this had no context modifications and was cleaned with 70% ethanol prior to training and testing for each animal. (2) Neutral context: this had six 2-inch white strips of tape placed on each wall of the darkened compartment at ~2-inch intervals and was cleaned with a citrus scented surface cleaner. Fiber photometry and electrophysiological experiments used IA chambers with the same modifications, but had higher walls to accommodate head-stage equipment.

IA discrimination test: Prior to experimentation, rats were handled for 3 min daily and habituated to the procedure room for 1 h over a 1-week period. During training (day 1), rats were placed in the enclosed, brightly-lit compartment of the aversive context and briefly allowed to acclimate (~10 s). During this time, the retractable door remained opened to allow free exploration of the entire apparatus. Upon entry into the darkened compartment, the door was closed to prevent the rat from returning to the brightly-lit compartment. Twenty seconds later, rats received a single inescapable footshock (0.8-mA, 1-s duration). Rats remained in the darkened compartment for an additional 20 s and were then removed. On day 3, rats were placed in the brightly-lit compartment of either the original aversive context or a neutral context (in a pseudo-randomized way), with the stainless-steel door retracted. The latency (in seconds) of the rat to cross into the darkened compartment was measured and used as an index of retention, with a

maximum latency of 600 s. After this test, rats were immediately transferred to an adjacent laboratory room for testing in the IA chamber equipped with the other context.

IA generalization test: Rats were handled prior to experimentation, as described above. During training (day 1), rats were placed in the brightly-lit compartment of the IA neutral context, with the stainless-steel door retracted, and allowed to explore the entire chamber for 1 min. Rats were then transferred to an adjacent laboratory room, where they were placed into the brightly-lit compartment of the aversive context, prior to being allowed entry into the darkened compartment, door closure, and subsequent footshock. To bias rats toward generalization, a longer shock duration (0.8 mA, 2 s duration) was used.[1, 78] After the footshock, rats were left in the dark compartment for 20 s prior to removal and optical manipulation. On day 3, retention testing was conducted by first placing rats in the lit compartment of the neutral context chamber. The latency of the rat crossing into the darkened compartment was measured. Immediately following, rats were transferred to the aversive context and similarly tested for latency to enter the dark compartment.

Optogenetics. Rats bearing ChR2 and mCherry control counterparts received 473-nm laser light (OptoEngine) pulses at 20 Hz (5-ms pulse width; Master-9 pulse generator). Halorhodopsin (Halo) and control animals received constant 561-nm laser light (Laser Century). Laser power for all experiments was adjusted to deliver ~10-mW power at the tip of the implanted fiber optic, which has been reported to be sufficient to activate opsins within a 0.46-mm radius sphere below the termination of the fiber tip.[79, 139] Optogenetic manipulations following IA training were performed in rats' home cages.

Neurophysiological recordings and analyses. A multielectrode recording system (Plexon) was used for neuronal ensemble recordings. In each rat, the common average reference was used for referencing, preserving 16 electrodes per animal. The Plexon Off-Line Sorter program was used for neuronal preprocessing. Spike activity was analyzed for all cells that fired at rates above 0.1 Hz. Principal component (PC) analysis and waveform shape were used for manual spike sorting. Units selected for additional analyses had (1) consistent waveform,[140] (2) distinct clustering in PC space, and (3) a refractory period of <2 ms. Analysis of neuronal activity was performed using custom routines in MATLAB. We constructed peri-event time histograms around latencies of rats to enter the darkened compartment of neutral and aversive IA contexts, from firing rate, with a bin size of 1 s and smoothed via a moving average with a span of 10 (smooth.m). Neuronal modulation was calculated by comparing firing rates or LFPs in the epoch before versus immediately after footshock or light-to-dark transitions using a Wilcoxon rank sum test; $p < 0.05$ was considered significant. Time-frequency spectrograms were calculated by taking the inverse fast Fourier transform (FFT) of the convolution of a FFT LFP power spectrum and a set of complex Morlet wavelets, as described in detail in our prior work.[89] Values from each electrode were included across four animals and compared by linear mixed-effect models accounting for effects across animals.

for verification of opsin functionality, rats bearing either dual viral preparation for excitation or inhibition of the rPL–BST pathway underwent neuronal recording procedures. At least 4 w after viral microinjection, rats underwent stereotaxic surgery for

the implantation of a 16-wire microelectrode with optical fiber, or “optrode” (MicroProbes for Life Science). Rats were anesthetized by IP injection of ketamine (100 mg/kg) and xylazine (10 mg/kg). Supplementary injections of ketamine (30 mg/kg) were given as needed. For optrode implantation, the scalp was retracted, and the skull was leveled between bregma and lambda. A craniotomy was made above the avBST of the right hemisphere. An additional hole for a single skull screw was made for connecting to a ground wire.

The optrode was slowly lowered (0.1 mm/min) into the dorsal-most aspect of the avBST. Neuronal recordings were made using a multielectrode recording system (Plexon). To determine whether regions were modulated by photoillumination, we recorded with the following parameters: 0–10 min, no laser; 10–20 min, 473-nm laser pulsed at 20 Hz with 10% duty cycle (for ChR2) or 561-nm laser (for Halo); 20–30 min, with the laser turned off. The optrode was then advanced ventrally by 0.3 mm three times, for a total of four recordings. After the recording session, the optrode was removed, and animals were perfused for histology.

Fiber photometry. Synapse software controlling an RZ10x lock-in amplifier (Tucker-Davis Technologies) was used to acquire fiber photometry data. LEDs (465-nm and 405-nm) were used as light sources for illumination of GCaMP8s to record Ca^{2+}-dependent and isosbestic changes, respectively. Light intensities were set to obtain ~50 µW power at the tips of optic fibers. Experimental timestamps (e.g., avoidance latency, footshock) were acquired using transistor-transistor logic pulses generated by the recording apparatus and manually verified via inspection of video recording. Raw data were transferred to Z-Score dF/F values and aligned to experimental timestamps by

using the open-source analysis software, pMAT.[141] Further analyses of fiber photometry data were performed using custom routines in MATLAB.

Histology and tissue processing. Upon completion of experiments, rats were anesthetized with pentobarbital (Fatal Plus; 150 mg/kg, IP) and perfused with 100 mL 0.9% NaCl, followed by 660 mL of ice-cold 4% paraformaldehyde (PFA), at a rate of 55 mL/min. Brains were then harvested and postfixed in 4% PFA at 4°C for 6 h and placed in cryoprotectant (20% sucrose/KPBS) for an additional 18 h. Coronal sections (30 µm) were collected in a 1:5 series on a sliding microtome (Leica). All sections were stored in a cryoprotectant solution at -20°C. Verification of viral expression and the placement of optical probes was performed by visualization of mCherry expression under epifluorescence with a compound light microscope (Leica) and confirmed based on cytoarchitectonic characteristics for the region of interest.[59] Rats with incorrect placement of virus or optic probes were excluded from subsequent analyses.

Hybridization histochemistry. In situ hybridization was performed using RNAscope (Advanced Cell Diagnostics).[142] Probes targeting GAD1 (ACDBio catalog #316401), vGlut2 (ACDBio catalog #317011), and mCherry (ACDBio catalog #513201) were obtained, and hybridization was performed using RNAscope Fluorescent Multiplex v2 (Advanced Cell Diagnostics). Visualization was performed using fluorophores (Fluorescein, Cyanine 3, Cyanine 5) with fluorescent signal enhancement using a modified tyramide signal amplification method (Perkin-Elmer). Slides were

counterstained with DAPI and cover slipped with ProLong Gold antifade reagent (Thermo Fisher).

Immunohistochemistry. Free-floating sections of coronal tissue were used for the localization of antigens. Primary antisera raised against anti-GFP (rabbit polyclonal; Thermo Fisher), mCherry (chicken polyclonal; Abcam), or c-Fos (rabbit polyclonal; raised against residues 4–17 of rat Fos protein; synthesized by J. Rivier and provided by P. Sawchenko, Salk Institute for Biological Studies, San Diego, CA) were visualized with goat anti-rabbit (Alexa 488; Thermo Fisher), goat anti-chicken (Alexa 555; Thermo Fisher) or goat anti-mouse (Alexa 680; Thermo Fisher), respectively.

Statistics. Plasma levels of stress hormones were analyzed using a repeated measure two-way ANOVA with blood collection time points (0, 10, 30, 60, 90 min) as the within-subjects variable and optogenetic treatment as the between-subjects factor. Post hoc pairwise comparisons using Fisher's least significant difference were used when appropriate. Integrated hormone levels (i.e., AUC) were analyzed using an unpaired t-test. Avoidance latency data were analyzed with a repeated measures ANOVA, with context and optogenetic treatment as factors. PC3, delta power (1–4 Hz), and theta power (4–8 Hz) were compared between novel and training contexts using linear-mixed effects models (*lmer)* in R, accounting for effects across animals. All main analyses were considered significant at $p < 0.05$. Details of statistical information for all the comparisons are found in *Statistical Analyses*.

STATISTICAL ANALYSES

Fig.	Description	Test	Statistic	Fisher's LSD post hoc comparison	Post hoc p value	Signif.
1H	%FG+/Fos+ neurons in rPL	t-test	t(4) = 3.96<br>$p = 0.017$			**
2G	CORT time series	RM ANOVA	Time<br>F(4, 96) = 51.16<br>$p < 0.0001$			****
			Virus<br>F(1, 24) = 4.63<br>$p = 0.042$			*
			Time X Virus<br>F(4, 96) = 1.45<br>$p = 0.225$			ns
2H	CORT AUC	t-test	t(24) = 2.26<br>$p = 0.033$			*
2I	IA latency	RM ANOVA	Context<br>F(2, 48) = 21.07<br>$p < 0.0001$			****
			Virus<br>F(1, 24) = 1.47<br>$p = 0.23$			ns
			Context X Virus<br>F(2, 48) = 3.92<br>$p = 0.027$	mCherry (D1) vs HALO (D1)	0.34	ns
				mCherry(D3-) vs Halo (D3-)	0.02	*
				mCherry (D3+) vs Halo (D3+)	0.90	ns
2J	Discrimination index	t-test	t(24) = 2.64<br>$p = 0.014$			*
2K	AM/PM CORT values	RM ANOVA	ADX Status<br>F(1, 22) = 0.23<br>$p = 0.639$			ns
			Time<br>F(1, 22) = 16.64<br>$P = 0.0005$			***
			ADX Status X Time<br>F(1, 22) = 2.58<br>$p = 0.12$			ns

2L	CORT post-restraint stress	t-test	t(22) = 18.30 $p < 0.0001$			****
2M	IA latency	RM ANOVA	Context F(2, 42) = 16.44 $p < 0.0001$			****
			Virus/ADX F(2, 21) = 2.65 $p = 0.095$			ns
			Context X Virus/ADX F(4, 42) = 3.45 $p = 0.016$	mCherry-Sham (D3-) vs HALO-ADX (D3-)	0.004	**
				mCherry-ADX (D3-) vs HALO-ADX (D3-)	0.002	**
				mCherry-Sham (D3-) vs mCherry-ADX (D3-)	0.96	ns
2N	Discrimination index	One-way ANOVA	F(2, 21) = 10.63 $p = 0.0006$	mCherry-Sham vs mCherry-ADX	0.51	ns
				mCherry-Sham vs HALO-ADX	0.001	**
				mCherry-ADX vs HALO-ADX	0.006	**
3B	IA latencies	Paired t-test	t(15) = 2.45 $p = 0.026$			*
3K	AUC latencies	RM ANOVA	10 s Pre – 10 s Post F(1, 4) = 44.32 $p = 0.0026$			**
			D1-D3 F(1, 4) = 0.06 $p = 0.81$			ns
			Interaction F(1, 4) = 29.68 $p = 0.0055$	D1_Pre vs D3_Pre	0.046	*
				D3_Pre vs D3_Post	0.0085	**

4G	PCA	LMER	PC1 ~Training vs Novel F(1, 78) = 1.80 $p = 0.19$	ns
			PC2 ~Training vs Novel F(1, 78) = 0.33 $p = 0.59$	ns
			PC3 ~Training vs Novel F(1, 78) = 0.03	*
4J	rPL Delta PSD	LMER	Context F(1, 218) = 96.67 $p < 0.0001$	****
			2s Pre – 2 s Post F(1, 217) = 57.34 $p < 0.0001$	****
			Interaction F(1, 217) = 39.91 $p < 0.0001$	****
4K	rPL Theta PSD	LMER	Context F(1, 219) = 52.96 $p < 0.0001$	****
			2 s Pre – 2 s Post F(1, 217) = 17.49 $p < 0.0001$	****
			Interaction F(1, 217) = 19.76 $p < 0.0001$	****
5E	CORT time series	RM ANOVA	Time F(5, 105) = 48.91 $p < 0.0001$	****
			Virus F(1, 21) = 0.002 $p = 0.96$	ns
			Time X Virus F(5, 105) = 0.46 $p = 0.81$	ns
5F	CORT AUC	t-test	t(21) = 0.20 $p = 0.85$	ns

5G, H, I	IA latency	RM ANOVA	Context F(5, 105) = 16.17 *p* < 0.001			***
			Virus F(1, 21) = 1.20 *p* = 0.29			ns
			Context X Virus F(5, 105) = 2.46 *p* = 0.037	mCherry(D3-) vs mCherry(D3+)	*p* = 0.10	ns
				ChR2(D3-) vs ChR2(D3+)	*p* < 0.001	***
				mCherry(D28-) vs mCherry (D28+)	*p* = 0.74	ns
				ChR2(D28-) vs ChR2(D28+)	*p* = 0.037	*
5G	Discrimination index D1	t-test	t(21) = 0.61 *p* = 0.547			ns
5J	Discrimination index D3	t-test	t(21) = 2.37 *p* = 0.028			*
5J	Discrimination index D28	t-test	t(21) = 1.53 *p* = 0.14			ns
S5	IA latency	RM ANOVA	Context F(2, 22) = 21.43 *p* < 0.001			ns
			Drug F(1, 11) = 7.11 *p* = 0.022	Saline(D3-) vs CORT (D3-)	*p* = 0.024	*
			Context X Drug F(2, 22) = 3.06 *p* = 0.067			ns
S7D	Entry in 3-chamber apparatus	RM ANOVA	Context F(1, 37) = 0.50 *p* = 0.48			ns
			5 s Pre – 5 s Post F(1, 37) = 7.50 *p* = 0.009	Light entry Pre vs Post	*p* = 0.20	ns
				Dark entry Pre vs Post	*p* = 0.008	**
			Interaction F(1, 37) = 0.35 *p* = 0.56			ns
S8F	Fos counts in rPL	t-test	t(7) = 3.28 *p* = 0.007			***

S8I, J	IA latency	RM ANOVA	Context F(3, 45) = 37.55 *p* = 0.001			ns
			Drug F(1, 15) = 3.99 *p* = 0.06			ns
			Context X Drug F(3, 45) = 3.08 *p* = 0.037	hm3dq-Sal(D1-) vs hm3dq-CNO(D1-)	*p* = 0.59	ns
				hm3dq-Sal(D1+) vs hm3dq-CNO(D1+)	*p* = 0.25	ns
				hm3dq-Sal(D3-) vs hm3dq-CNO(D3-)	*p* = 0.037	*
				hm3dq-Sal(D3+) vs hm3dq-CNO(D3+)	*p* = 0.42	ns
S8I (right)	Discrimination index	t-test	t(15) = 1.43 *p* = 0.17			ns
S8K	IA latency	RM ANOVA	Context F(1, 15) = 0.112 *p* = 0.742			ns
			Drug F(1, 15) = 0.16 *p* = 0.69			ns
			Context X Drug F(1, 15) = 0.001 *p* = 0.98			ns
S8L	Discrimination index D3	t-test	t(15) = 1.54 *p* = 0.15			ns
S8L	Discrimination index D28	t-test	t(15) = 0.005 *p* = 0.99			ns
S9L (left)	IA latency	RM ANOVA	Context F(1, 16) = 2.42 *p* = 0.139			ns
			Virus F(1, 16) = 0.03 *p* = 0.86			ns
			Context X Virus F(1, 16) = 0.21 *p* = 0.65			ns
S9L (right)	Discrimination index	t-test	t(16) = 0.465 *p* = 0.648			ns
